# Analyzing Molecular Traits of H9N2 Avian Influenza Virus Isolated from a Same Poultry Farm in West Java Province, Indonesia, in 2017 and 2023

**DOI:** 10.12688/f1000research.150975.1

**Published:** 2024-06-04

**Authors:** Muhammad Ade Putra, Amin Soebandrio, I Wayan Teguh Wibawan, Christian Marco Hadi Nugroho Nugroho, Ryan Septa Kurnia, Otto Sahat Martua Silaen, Rifky Rizkiantino, Agustin Indrawati, Okti Nadia Poetri, Desak Gede Budi Krisnamurti

**Affiliations:** 1 Master of Animal Biomedical Sciences, School of Veterinary and Biomedical, IPB University, Bogor, West Java, 16680, Indonesia; 2 Department of Microbiology, Faculty of Medicine, University of Indonesia, Jakarta, Jakarta, 10320, Indonesia; 3 Division of Medical Microbiology, School of Veterinary Medicine and Biomedical Sciences, IPB University, Bogor, West Java, 16680, Indonesia; 4 Animal Health Diagnostic Unit, PT. Medika Satwa Laboratoris, Bogor, West Java, 16166, Indonesia; 5 Division of Central Laboratory and Disease Research Center, Technology and Research Development, Central Proteina Prima (CP Prima) Inc., Tangerang, Banten, 15560, Indonesia; 6 Department of Medical Pharmacy, Faculty of Medicine, University of Indonesia, Jakarta, Jakarta, 10430, Indonesia

**Keywords:** avian influenza, characterization, docking, H9N2, Indonesia, Mutation

## Abstract

**Background:**

Indonesia is one of the countries that is endemic to avian influenza virus subtype H9N2. This study aims to compare the molecular characteristics of avian influenza virus (AIV) subtype H9N2 from West Java.

**Methods:**

Specific pathogen-free (SPF) embryonated chicken eggs were used to inoculate samples. RNA extraction and RT–qPCR confirmed the presence of H9 and N2 genes in the samples. RT–PCR was employed to amplify the H9N2-positive sample. Nucleotide sequences were obtained through Sanger sequencing and analyzed using MEGA 7. Homology comparison and phylogenetic tree analysis, utilizing the neighbor-joining tree method, assessed the recent isolate’s similarity to reference isolates from GenBank. Molecular docking analysis was performed on the HA1 protein of the recent isolate and the A/Layer/Indonesia/WestJava-04/2017 isolate, comparing their interactions with the sialic acids Neu5Ac2-3Gal and Neu5Ac2-6Gal.

**Results:**

RT–qPCR confirmed the isolate samples as AIV subtype H9N2. The recent virus exhibited 11 amino acid residue differences compared to the A/Layer/Indonesia/WestJava-04/2017 isolate. Phylogenetically, the recent virus remains within the h9.4.2.5 subclade. Notably, at antigenic site II, the recent isolate featured an amino acid N at position 183, unlike A/Layer/Indonesia/WestJava-04/2017. Molecular docking analysis revealed a preference of HA1 from the 2017 virus for Neu5Ac2-3Gal, while the 2023 virus displayed a tendency to predominantly bind with Neu5Ac2-6Gal.

**Conclusion:**

In summary, the recent isolate displayed multiple mutations and a strong affinity for Neu5Ac2-6Gal, commonly found in mammals.

## Introduction

Avian influenza is an acute viral infectious disease that can affect all types of birds of any age. Based on the differences in hemagglutinin (H1–18) and neuraminidase (N1–N10) components, avian influenza is divided into several subtypes. ^1^^,^^2^ Each subtype has varying levels of pathogenicity, ranging from low pathogenic avian influenza (LPAI) to high pathogenic avian influenza (HPAI). However, both LPAI and HPAI can affect bird health. ^3^ The presence of gene mutations through antigenic drift and antigenic shift can result in a change in symptoms. Initially, LPAI may not cause significant harm, but with these mutations, it can become highly detrimental to the poultry industry. ^4^

AIV is an RNA virus that consists of eight segments, each encoding viral protein genes. On the other hand, this virus is also more prone to mutation than DNA viruses. ^5^ Diagnosis using polymerase chain reaction (PCR) and sequencing of the viral genome is necessary to determine the homology between the vaccine virus and the circulating field virus. ^6^ This is because if the circulating virus has low homology with the vaccine, it can result in the ineffectiveness of the vaccine due to the continued shedding of the virus. ^7^

In AIV, there is a crucial virus segment that needs to be characterized, which is hemagglutinin (HA), corresponding to the fourth segment of the AI genome. ^8^^,^^9^ HA functions at the early stage of infection by attaching the virus to the host receptor. Mutations in the HA gene can increase the virulence and pathogenicity of the virus. ^10^ Avian influenza infects its target host by initiating the recognition of the HA protein by cellular proteases from the target infected cell. Cellular proteases activate HA0, splitting it into two parts: HA1 and HA2. HA1 binds to Neu5Ac, while HA2 plays a role in fusion between the viral envelope and the host endosomal membrane. Without this recognition, the virus cannot infect the cell. ^11^

One subtype AIV that poses a threat to the poultry industry is the H9N2 subtype. ^12^ In countries affected by avian influenza outbreaks, such as China, the H9N2 subtype of AIV causes significant losses due to its high morbidity rate of up to 100% in infected layer chicken farms. ^13^ Infected layer chickens experience a decrease in appetite accompanied by a drastic decline in egg production. Viral infection is often exacerbated by secondary infections from bacteria or other viruses, resulting in high mortality among the infected layer chicken population. ^14^ H9N2 virus infection may not be immediately visible at onset, but the virus spreads rapidly through the shedding of feces or nasal discharge from birds. Due to these characteristics, rapid and accurate detection is crucial to identify the presence of AIV infection on a farm, enabling appropriate measures to be taken in addressing H9N2 virus infection. ^15^

West Java Province is a province in Indonesia that has several densely populated districts for layer chicken farming, including Bogor, Sukabumi, Cianjur, Ciamis, and several other districts. In 2017, one H9N2 isolate was successfully isolated and characterized, namely, strain A/Layer/Indonesia/WestJava-04/2017 (GenBank accession number MG957203), originating from a densely populated layer chicken farming area in West Java. ^16^ The high density of layer chicken farms in a region facilitates the easy spread and mutation of AIVs. Therefore, this study aims to isolate and characterize the H9N2 subtype AIV from the layer chicken farm where the A/Layer/Indonesia/WestJava-04/2017 strain originated. This study also describes the molecular changes in the HA gene using molecular docking after implementing the H9N2 vaccination program, which has been ongoing for six years.

## Methods

### Sample collection

Sampling was conducted at a commercial layer chicken farm in West Java Province, the original location of the AIV subtype H9N2 strain A/Layer/Indonesia/WestJava-04/2017 (GenBank Accession No. MG957203). After five years of vaccination with a homologous vaccine at the farm, a decrease in production and bird mortality occurred in February 2023. Samples consisting of brain, trachea, and oviduct organs pooled from deceased chickens.

### Virus isolation

The sample was multiplied using standard laboratory procedures. Tissue was combined with sterile phosphate-buffered saline from ThermoFisher (28348) pH 7.4 containing antibiotics (200 g/ml penicillin and 100 g/ml streptomycin) and then centrifuged at 1,000 g and 4 °C for fifteen minutes. The remaining supernatant was filtered through a 0.45 m membrane filter and injected intra-allantoically into 9-day-old SPF ECEs. The SPF ECEs were incubated at 37 °C for 48–72 hours, and daily monitoring of embryo mortality was performed. ^17^ After 72 hours of incubation, all ECEs, regardless of their viability, were subsequently preserved at a refrigerated temperature of 8 °C overnight and then harvested. The collected specimens consisted of allantoic fluid, which was subsequently utilized for conducting a rapid HA assay to promptly detect AIV in early stages. ^16^

### Viral RNA was identified using a reverse transcription-polymerase chain reaction (RT–PCR) assay

The allantoic fluid that was collected was further extracted using a total RNA mini kit reagent from Geneaid ^®^, Taiwan (RPD050). The RNA obtained was then analyzed for the presence of the H9N2 virus using the SensiFASTTM SYBR Lo-ROX Kit from Bioline ^®^, Taunton (BIO-94005). For the RT–qPCR assay, the primers H9-F: 5′-ATCGGCTGTTAATGGAATGTGTT-3′, H9-R: 5′-TGGGCGTCTTGAATAGGGTAA-3′ ^18^ and N2-F: 5′-CTCCAATAGACCCGTACTAT-3′, N2-R: 5′-CCTGAAGTCCCACAAAATAC-3′ ^19^ were utilized with the LongGene Q2000C (China). The thermal profile for gene amplification included two minutes of polymerase activation at 95 °C, which was followed by a total of 45 cycles of denaturation at 95 °C for 5 seconds, annealing at 60 °C for 10 seconds, and extension at 72 °C for 15 seconds. When the cycle threshold (Ct) value falls below 40, a positive result is indicated. In addition, RT–PCR assays were conducted to detect avian influenza subtype H9N2, Newcastle disease virus (NDV), and infectious bronchitis virus (IBV). RT–PCR was conducted according to the protocol used in a previous study. ^20^

### Sequencing

The confirmed HA of the H9 gene from RT–PCR was amplified for sequencing using the MyTaq One-Step RT–PCR kit from Bioline ^®^, Taunton (BIO-65049), and the primers HAp1-F: 5′-TCCACGGAAACTGTAGACACA-3′, HAp1-R: 5′-TTCTGTGGCTCTCTCCTGAAA-3′ and Hap2-F: 5′-AGGCCTCTTGTCAACGGTTT-3′, Hap2-R: 5′-CCAACGCCCTCTTCACTTTA-3′ were used. ^21^ The Sanger sequencing method was performed by First BASE Laboratories, Malaysia, which separated the PCR products using electrophoresis and purified the desired band for sequencing. Using Bioedit v.7 ( https://bioedit.software.informer.com/7.0/), the nucleotide and amino acid sequences of a recently isolated strain’s HA gene were determined, and ClustalW was used for alignment. A modern virus phylogenetic tree was constructed using MEGA v 7.0 ( https://www.megasoftware.net/), the neighbor-joining method, and 1,000 alignment repetitions. The genetic distance between isolates and the topology of the phylogenetic tree were used to designate strains. ^22^

A comparison was made between the recent study isolate, a previous isolate (A/Layer/Indonesia/WestJava-04/2017, GenBank Accession No. MG957203) and other H9N2 isolates from GenBank. Several key regions were investigated in this investigation, including the receptor-binding sites (RBS) on the left and right edges of the binding pocket, as well as the cleavage site. In addition, the correlation between particular amino acid residues (54, 80, 106, 109, 113, 123, 125, 129, 130, 135, 137, 146, 147, 149, 150, 152, 164, 165, 178, 179, 182, 183, 188, 189, 194, and 216) and H9N2 virus antigenicity was analysed. The amino acid sequence of the HA gene from the most recent H9N2 virus was uploaded to http://www.cbs.dtu.dk/services/NetNGlyc/to evaluate potential N-glycosylation sites. ^21^

### Molecular Docking of Protein HA1 (2017 and 2023 isolates) with Ligands Neu5Ac2-3Gal and Neu5Ac2-6Gal

The simulation was conducted on a computer hardware system with the following specifications: 8.00 GB RAM and an Intel ^®^ Core i5-2520 M CPU with a clock speed of 2.50 GHz. The software used in the study included Google Chrome v114.0.5735.199 for accessing the SwissDock website, which served as the protein–ligand docking server and ran automatically ( http://swissdock.ch/docking). Additionally, BIOVIA Discovery Studio Visualizer v21.1.0.20298 ( https://discover.3ds.com), Chimera v1.17.3 ( https://www.cgl.ucsf.edu/chimera/download.html), AutoDock Tools v1.5.7 ( https://autodock.scripps.edu/), PyMol v2.5.2 ( https://pymol.org/), and LigPlot+ v2.2.8 ( https://www.ebi.ac.uk/thornton-srv/software/LigPlus/download.html) were used for data analysis and visualization.

The simulation begins with the creation of a 3D model of the HA1 protein from the AIV, specifically the recent study isolate (HA1-2023) and the A/Layer/Indonesia/WestJava-04/2017 strain (HA1-2017). This is achieved using the PHYRE2 Protein Fold Recognition Server ( http://www.sbg.bio.ic.ac.uk/). The ligands used in the simulation were obtained from https://pubchem.ncbi.nlm.nih.gov/. They are Neu5Ac2-3Gal (compound CID: 13832708) and Neu5Ac2-6Gal (compound CID: 53262334).

The docking was performed using the automated server facility on SwissDock, where the uploaded documents were in PDB format (.pdb) for the protein acting as the receptor and SYBYL MOL2 format (.mol2) for the molecules acting as ligands. Docking was carried out for the protein HA1-2017 with the Neu5Ac2-3Gal ligand, HA1-2017 with the Neu5Ac2-6Gal ligand, HA1-2023 with the Neu5Ac2-3Gal ligand, and HA1-2023 with the Neu5Ac2-6Gal ligand. Only models with ligands attached to the Leu216 residue region are observed and selected for further analysis.

The selection of the residue is based on the fact that the amino acid at position 216 determines the virus’s tendency to infect mammalian cells dominated by Neu5Ac2-6Gal or avian cells dominated by Neu5Ac2-3Gal. ^20^ The docking results were downloaded in the Chimera Web Data format (.xml) and run in Chimera software v1.17.3 to select the best docking model. The best model was chosen based on the Gibbs free energy (ΔG) value, where ΔG represents the stability parameter of the bond formed between the protein and the ligand. A more negative value indicates a more stable bond. The protein–ligand interactions are then evaluated, observing hydrogen bonding, hydrogen bond distances (Å), interacting residues and functional groups, and residues that interact with the ligand noncovalently. ^23^

### Nucleotide sequence accession numbers

The partial CDS of the HA gene in this study was deposited in GenBank and received the accession number OR243721.

## Results

### H9N2 subtype AI virus detection

**Figure 1.**
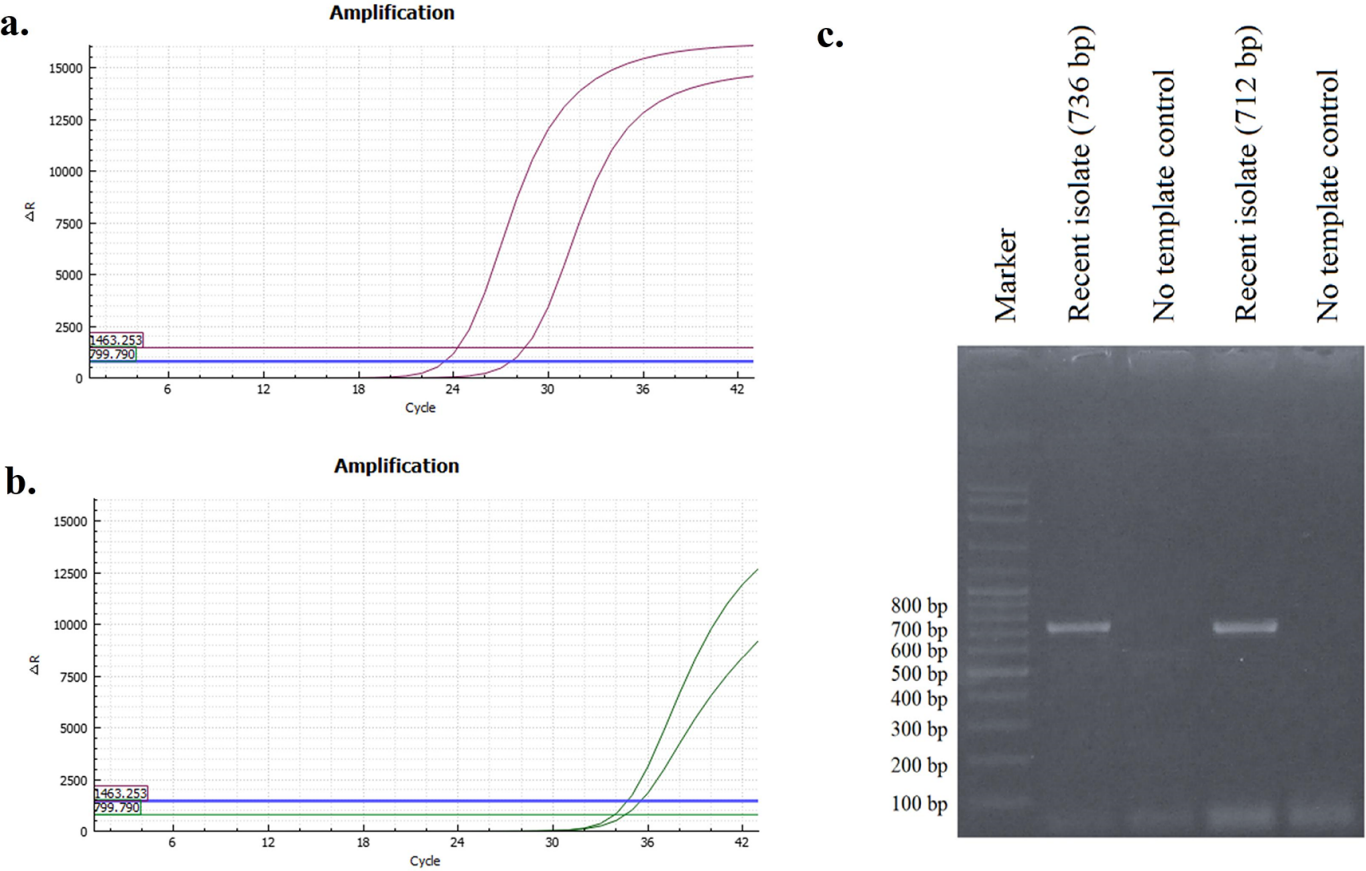
Curves of H9 (a) and N2 (b) RT–qPCR results. The sample revealed single band (c).

The presence of viral RNA was identified by RT–qPCR specifically targeting H9 and N2. The results shown in Figure 1a and Figure 1b indicate that the recent isolate of this study is an AIV subtype H9N2. The H9 gene in a recent isolate was amplified with a Ct value of 28.50, while the N2 gene was amplified at a Ct value of 34.69. A positive result was determined when the Ct value was less than 40 and displayed an amplification curve resembling that of the positive control. Conversely, a negative result was determined if the Ct value was 45 or higher, and no amplification curve resembling the positive control was observed. The results with a Ct value between 40 and 45 were classified as indeterminate or dubious.

### Amplification of the Hemagglutinin (HA) gene

The results of HA gene amplification using primers HApar1 and HApar2 on a recent sample from this investigation are shown in Figure 1c. The electrophoresis results revealed the presence of DNA bands at the 736 bp (for HApar1) and 712 bp (for HApar2) sites. Marker 100-2000 bp is the size of the molecular marker displayed in the illustration.

### Amino acid analysis

The partial CDS of the HA gene from this study was deposited in GenBank, with the accession number OR243721 (A/chicken/Indonesia/MSL0123/2023). The amino acid sequence at the cleavage site of the recent isolate is PSRSSR↓GLF, while the receptor binding site has the motif PWTNTLY ( Table 1). Additionally, at position 217 on the left side of the receptor binding site (RBS), the recent isolate contained the amino acid M (methionine). It should be noted that this amino acid sequence is identical to A/Layer/Indonesia/WestJava-04/2017, which originated from the same farm.

**Table 1.** Amino acid analysis of important sites in the HA gene of H9N2 viruses.

A. Receptor-binding pockets, cleavage sites and antigenic site of recent isolate of H9N2 compared to A/Layer/Indonesia/WestJava-04/2017 *^a^*								
Virus	Lineage	Receptor binding site	Left-edge of binding pocket	Right-edge of binding pocket	Cleavage site	Antigenic site		Acession Number
						Site I	Site II	
A/Layer/Indonesia/WestJava-04/2017	h9.4.2.5	PWTNTLY	NGLMGR	GTSKA	PSRSSR↓GLF	SKP	DDL	MG957203
A/chicken/Indonesia/MSL0123/2023	h9.4.2.5	PWTNTLY	NGLMGR	GTSKA	PSRSSR↓GLF	SKP	DNL	OR243721

B. Potential N-glycosylation sites _2)_									
					HA1				
Virus	11–13 ^b^	123–125	127–202	178–180	188–190	200–202	280–282	287–289	295–297
A/Layer/Indonesia/WestJava-04/2017	NST	NVS	–	–	–	NRT	NTT	NVS	NCS
A/chicken/Indonesia/MSL0123/2023	NST	NVS	–	–	–	NRT	NTT	NVS	NCS

C Other mutations of recent isolate										
					HA1					
Virus	23	34	53	69	72	74	114	120	163	179
A/Layer/Indonesia/WestJava-04/2017	N	H	D	P	E	R	I	K	E	T
A/chicken/Indonesia/MSL0123/2023	D	Q	E	S	G	K	T	T	G	D

^a^ The receptor-binding site (RBS) includes residues at positions 92, 143, 145, 173, 180, 184, and 185. The left-edge of the binding pocket is located at positions 214-219, while the right-edge of the binding pocket is situated at positions 128-132. The cleavage site is positioned at positions 315-323. The antigenic site is divided into two regions: site I, which includes positions 125, 147, and 152, and site II, which encompasses positions 135, 183, and 216.

^b^ The arrangement of amino acid residues in the HA genes of AIV subtype H9N2.These residue placements are based on the H9 numbering system.

In a recent study, the antigenic site I of the HA gene in the recent sample displayed the same motif as A/Layer/Indonesia/WestJava-04/2017. At site I, amino acids S (serine), K (lysine), and P (proline) were present. For site II, the motif consisted of amino acids D (aspartate), N (asparagine), and L (leucine) at positions 135, 183, and 216. In contrast, A/Layer/Indonesia/WestJava-04/2017 exhibited D (aspartate) at position 183. The analysis of the HA gene in the recent sample included an examination of potential glycosylation sites (PGS). The HA1 gene segment revealed the NXT/S motif (where X represents any amino acid except proline) at positions 11-13 (NST), 123-125 (NVS), 200-202 (NRT), 280-282 (NTT), 287-289 (NVS), and 295-297 (NCS). There were also several other amino acid differences observed at positions 23, 34, 53, 69, 72, 74, 114, 120, 163, and 179 ( Table 1).

### Homology comparison

The results of the homology comparison are shown in Table 2. The recent isolate has a similarity of 95.84% with A/Layer/Indonesia/WestJava-04/2017, which was isolated from the same farm but in a different year. When analyzed against several representatives of the H9N2 subtype clades, the recent isolate has a similarity of approximately 72.24% with clade h9.1, 72.56% with clade h9.2, 74.72% with clade h9.3, and 81.80% with h9.4.1. These results are lower compared to the similarity of the recent isolate with viruses classified under clade h9.4.2. Based on homology comparison, a recent isolate, A/chicken/Indonesia/MSL0123/2023, belonged to subclade h9.4.2.5.

**Table 2.** Nucleotide sequence similarities between the current strain and several H9N2 isolates in GenBank.

No.	Strain	Accession No.	Clade	Similarity (%)
1	A/turkey/California/189/66	AF156390	h9.1	72.24
2	A/Shorebird/Delaware/9/96	AF156386	h9.2	72.56
3	A/Duck/Hong_Kong/Y439/97	AF156377	h9.3	74.72
4	A/Quail/Hong_Kong/G1/97	AF156378	h9.4.1	81.80
5	A/Chicken/Shanghai/F/98	AY743216	h9.4.2.1	86.05
6	A/guineafowl/HongKong/NT184/03	AY664674	h9.4.2.2	83.66
7	A/Chicken/Guangdong/SS/94	AF384557	h9.4.2.3	86.95
8	A/Duck/Hong_Kong/Y280/97	AF156376	h9.4.2.4	88.30
9	A/chicken/Henan/LY-36/2013	KF638574	h9.4.2.5	90.95
10	A/Layer/Indonesia/WestJava-04/2017	MG957203	h9.4.2.5	95.84
11	A/muscovy_duck/Vietnam/LBM719/2014	LC028176	h9.4.2.5	95.55
12	A/chicken/Guangdong/FZH/2011	JF715024	h9.4.2.6	84.21

### Phylogenetic tree

**Figure 2.**
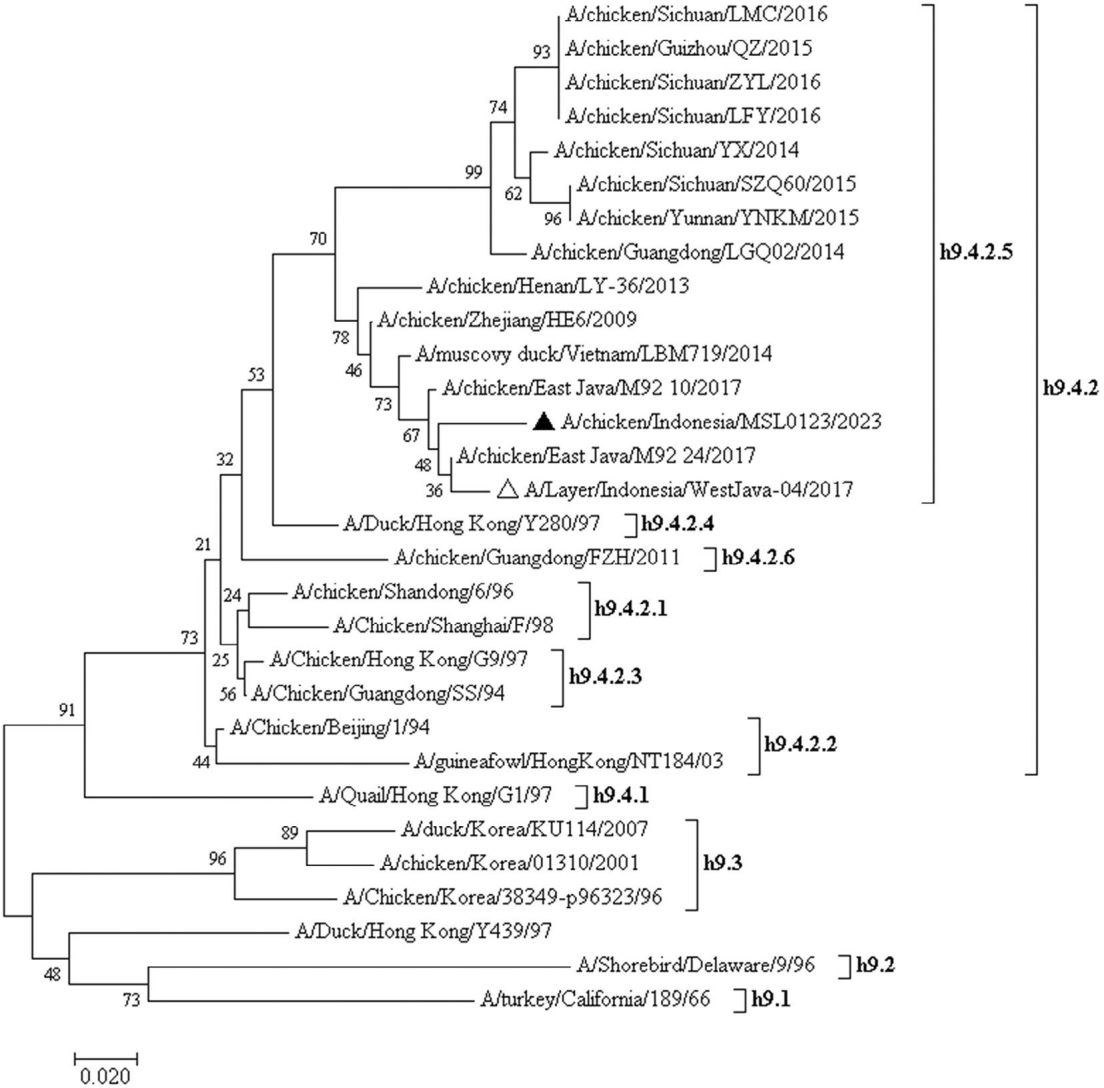
Phylogenetic tree of the hemagglutinin (HA) gene of the AIV H9N2 virus was constructed.

The phylogenetic tree showed that the samples were together with other isolates from Indonesia (A/Layer/Indonesia/WestJava-04/2017, A/chicken/East_Java/M92_10/2017, A/chicken/East_Java/M92_24/2017), Vietnam (A/muscovy_duck/Vietnam/LBM719/2014) and China (A/chicken/Zhejiang/HE6/2009, A/chicken/Henan/LY-36/2013, A/chicken/Guangdong/LGQ02/2014) so that these were included in China, Vietnam, and Indonesia (CVI) clades ( Figure 2). Recent viruses belonged to subclade h9.4.2.5, according to a phylogenetic study.

### Docking analysis

**Figure 3.**
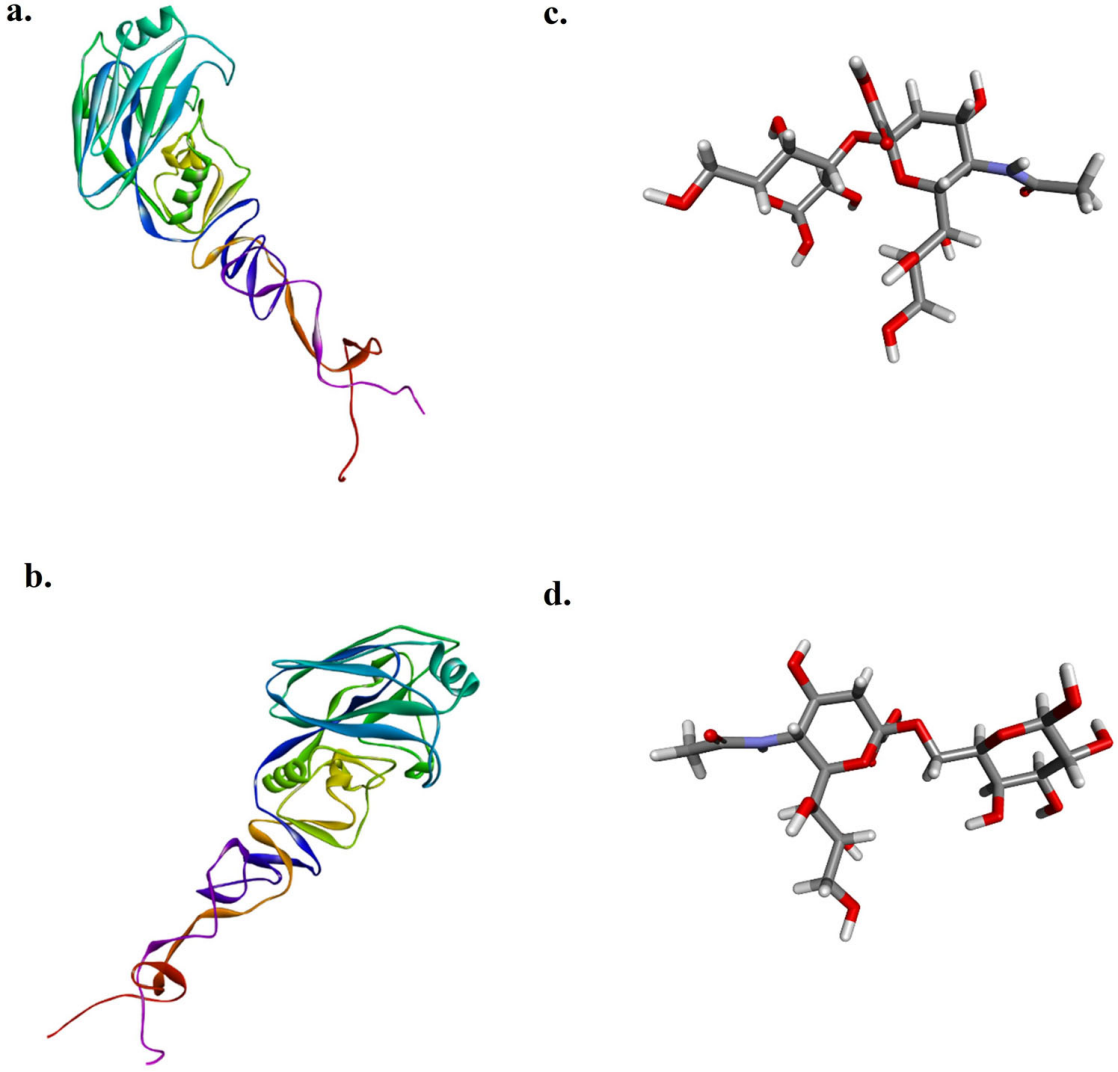
Modeled protein HA1 of H9N2 predicted by the Robetta server, visualized in Discovery Studio Visualizer.

The target HA1 proteins of AIV subtype H9N2 isolated in 2017 (HA12017) and 2023 (HA12023) were chosen for the study because of their importance in viral attachment for the docking analysis, as there were no 3D structures of the protein, and its modeling was carried out. After submission of their amino acid sequences on Phyre2, the modeled proteins obtained were downloaded and visualized in Discovery Studio Visualizer. The Neu5Ac2-3Gal (Sia3) and Neu5Ac2-6Gal (Sia6) ligands used in this study are shown in Figure 3.

**Figure 4.**
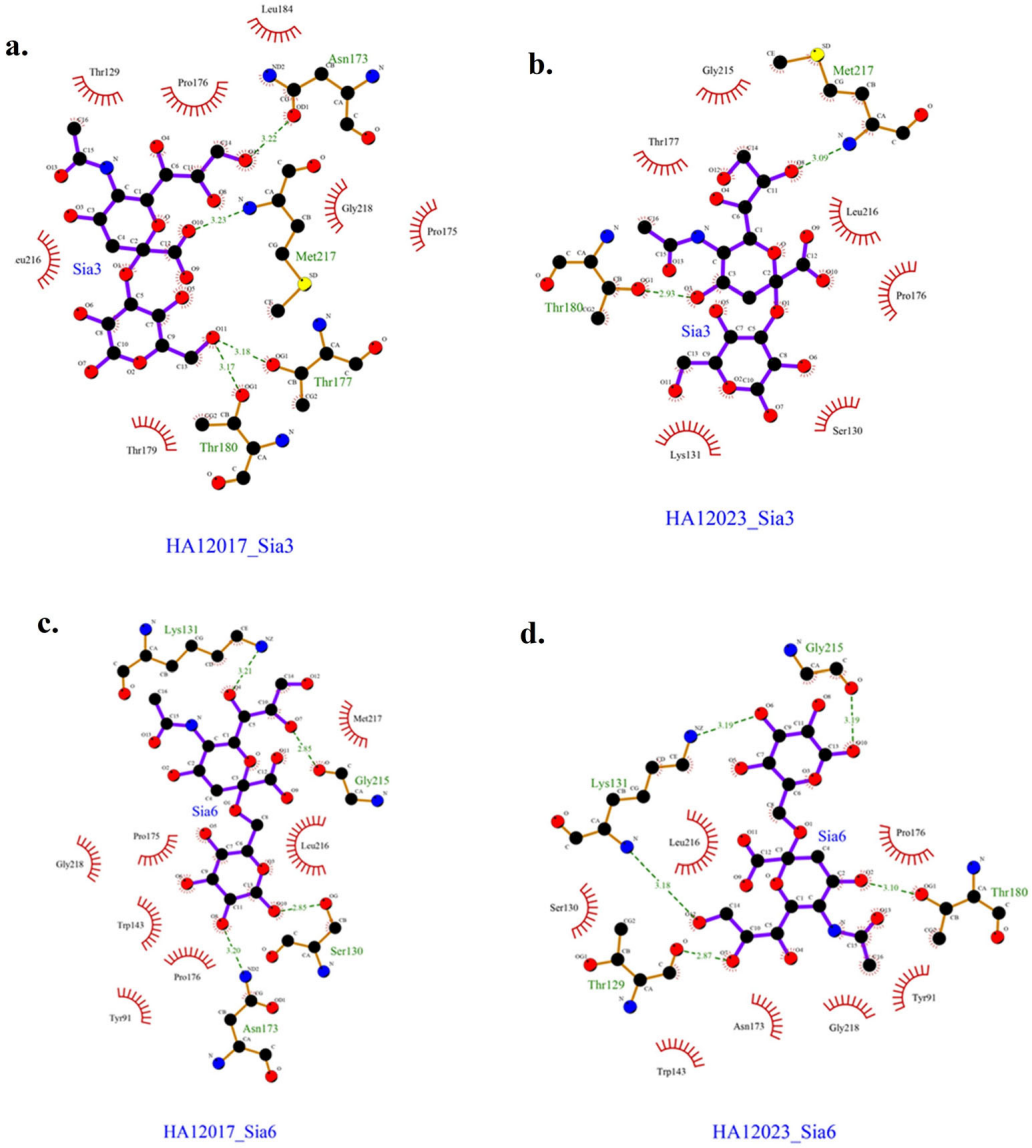
Interactions occurring in HA12017 and HA12023 with ligand Sia3 and Sia6.

Based on the docking results, the data obtained are presented in Table 3. The obtained results represent the best model with the most negative ∆G and the ligand positioned and bound near the Leu216 residue. The interactions between each protein and ligand are shown in Figure 4, with hydrogen bonding data and residues involved in hydrogen bonding interactions presented in Table 4. The interactions that occur can be either hydrogen bonding or nonbonding interactions that strengthen the ligand’s affinity with the protein.

**Table 3.** Docking results of proteins HA12017 and HA12023 with ligands Sia3 and Sia6 selected.

Protein	Ligand	Cluster	Cluster rank	Delta G ( ∆ G) (Kcal/mol)
HA12017	Sia3	2	0	-7.98304
HA12017	Sia6	11	0	-7.79904
HA12023	Sia3	2	0	-7.42221
HA12023	Sia6	8	0	-7.84408

**Table 4.** Hydrogen bonds and interacting residues.

Protein	Ligand	Hydrogen bond distance (Å)	Residues and functional groups that are binding	Residues that have nonbonding interactions with the ligand
HA12017	Sia3	3.22	Asn173 (O-O)	Thr129, Pro178, Leu184, Pro175, Gly218, Thr179, Leu216
		3.23	Met217 (O-N)	
		3.18	Thr177 (O-O)	
		3.17	Thr180 (O-O)	
HA12023	Sia3	2.93	Thr180 (O-O)	Thr177, Gly215, Leu216, Pro176, Ser130, Lys131
		3.09	Met217 (O-N)	
HA12017	Sia6	3.21	Lys131 (O-N)	Gly218, Pro175, Trp143, Pro176, Tyr91, Leu216, Met217
		2.85	Gly215 (O-O)	
		2.85	Ser130 (O-O)	
		3.20	Asn173 (O-N)	
HA12023	Sia6	3.18	Lys 131 (O-N)	Ser130, Leu216, Pro176, Tyr91, Gly218, Asn173, Trp143
		3.19	Lys 131 (O-N)	
		3.19	Gly215 (O-O)	
		3.10	Thr180 (O-O)	
		2.87	Thr129 (O-O)	

## Discussion

While numerous genes are involved in avian influenza pathogenesis, the HA surface glycoprotein remains critical. ^24^ Hemagglutinin regulates the parameters that influence the virulence and pathogenicity of avian influenza virus. The amino acid makeup of the HA gene includes the cleavage site, receptor binding site (RBS), antigenic site, and whether or not glycosylation sites surround the receptor binding sites. ^25^

The cleavage site, which is located between the HA1 and HA2 subunits of the HA gene, is critical in determining the pathogenicity of AIV. ^26^ Clades H9.1, H9.2, and H9.3 have a single arginine (R) residue at the cleavage site, whereas clade H9.4 has two arginine (R) residues (dibasic), and other isolates have three arginine (R) residues (tribasic) at this site. ^21^ Viral evolution is responsible for the formation of H9N2 virus variants with several residues (dibasic/tribasic) at the cleavage site. ^27^ Unless accompanied by additional infections, the presence of dibasic/tribasic residues at the cleavage site in H9N2 viruses does not significantly increase pathogenicity in the host. ^27^

The presence of an RSSR motif at a virus’s cleavage site influences viral propagation, host tissue tropism, and viral pathogenicity. ^28^ This specific motif, PSRSSR↓GLF, was previously identified in A/Layer/Indonesia/WestJava-04/2017, A/Layer/Indonesia/WestJava-06/2017, A/Layer/Indonesia/WestJava-09/2017, A/Layer/Indonesia/WestJava-13/2017, A/Layer/Indonesia/WestJava-14/2017, A/Layer/Indonesia/WestJava-15/2017, and A/Layer/Indonesia/CentralJava-01/2017. ^16^ The H9.4.2.5 clade of the H9N2 virus has a PSRSSRGLF pattern in cleavage, indicating that it is a low pathogenic avian influenza (LPAI) virus. This motif selectively responds to the trypsin enzyme, which is mostly released by the digestive and respiratory systems, limiting the breadth of the infection. ^29^

The current isolate in this study has the PWTNTLY amino acid motif of the receptor-binding site (RBS). The isolate A/Layer/Indonesia/WestJava-04/2017 is the same. RBS position 180 has been reported to influence the amount of bond affinity to sialyl-2,6-galactose (human cell receptor). Threonine (T) at position 180 had the highest binding affinity. ^30^ The motif of the H9.4.2.5 subclade is amino acid M (methionine) at position 217 on the left side of the RBS. ^31^ In Indonesia, this motif was previously found in isolates A/Layer/Indonesia/WestJava-04/2017, A/Layer/Indonesia/WestJava-06/2017, A/Layer/Indonesia/WestJava-09/2017, A/Layer/Indonesia/WestJava-13/2017, A/Layer/Indonesia/WestJava-14/2017, A/Layer/Indonesia/WestJava-15/2017, and A/Layer/Indonesia/CentralJava-01/2017. ^16^

Threonine (T) is found at position 129 on the right side of the receptor binding site (RBS), and lysine (K) is found at position 131. The H9.4.2 subclade is the only subclade with these unique I129T and R131K amino acid changes. ^21^ A single modification to an amino acid within the receptor binding pocket can have a major impact on the AI virus’s ability to infect and disseminate. This element is critical in influencing AIV host diversity. ^32^ Notably, changed receptor binding avidity of H9N2 viruses, including higher binding to human-like receptors, results in antigenic diversity in AIVs, raising the possibility of spreading zoonotic agents. ^33^

The antigenic site motif detected in the sample was similar to that of A/Layer/Indonesia/WestJava-04/2017 and other Indonesian isolates. The residue at position 216 inside this antigenic region is important in receptor-binding interactions with the host. Human influenza viruses that contain the amino acid L216 preferentially bind to Neu5Ac2-6Gal receptors, whereas AIVs that contain the amino acid Q216 preferentially bind to Neu5Ac2-3Gal receptors. ^34^ Additionally, the change from Q216L can improve the virus’s capacity for infecting people. ^35^

H9N2 pathogens are prevalent and have rarely caused severe illnesses in people. Although massive chicken vaccination has been beneficial in suppressing H9N2, it may have also contributed to the virus’s evolutionary alterations. ^36^ As a result, it is critical to track the evolution of circulating H9N2 strains and assess their potential dangers to veterinary medicine and public health. We discovered numerous mutations that could have been impacted by vaccination in this study.

Regarding the eleven amino acid variations among a recent isolate and A/Layer/Indonesia/WestJava-04/2017, position 183 was discovered to alter H9N2 avian influenza infection. Residue N in this location encodes the preference for human-like receptors. One change in amino acids at this position significantly impacted H9N2 antigenicity, as measured by monoclonal antibodies and antisera. ^37^ in vivo studies demonstrated that changes influenced viral replication and transmission of H9N2 in chickens. D183-containing viruses were able to multiply in the lungs of infected hens. Variations in antigenic site II locations 183 and 216 decrease viral interactions with epitope-specific antibodies and can result in mutant virus escape. ^16^^,^^38^

The possibility of a glycosylation site (PGS) pattern in the sample isolate matched those observed in H9.4.2.5, including A/Layer/Indonesia/WestJava-04/2017 and other Indonesian isolates. A viral method involves changing the amino acid sequence at the glycosylation site to conceal or reveal the antigenic area, preventing it from being recognized by the immune system. The addition of PGS to the sample at residue positions 295-297 may improve the virus’s ability to evade neutralization by host antibodies. ^31^^,^^39^

According to phylogeography, the H9 subtype of AIV can be separated into two basic lineages: American and Eurasian. ^40^ The H9 subtype is divided into four clades based on HA gene segments. ^41^ The H9.1 clade is derived from the A/turkey/Wisconsin/1/1966 virus found in bird populations on the continent of North America. The H9.2 clade of the American lineage is linked to the A/quail/Arkansas/29209-1/93 strain from the Arkansas region. The Eurasian lineage, on the other hand, is composed of two primary gene lines: Y439-H9.3 (A/duck/HongKong/Y439/1997) and G1-H9.4 (A/Quail/Hong Kong/G1/1997). The Y439 lineage, which has spread in wild bird species across Asia, Africa, and Europe, is likely to be the closest to the origin of the AI virus. The G1 lineage has additionally been reported, which is most typically found in commercial poultry and live poultry markets. ^42^ The H9.4 clade has been divided into two subclades: H9.4.1 and H9.4.2. Since 2013, the H9.4.2 subclade of the H9N2 AI virus has been discovered in China. ^13^ Based on a genetic distance analysis, the recent sample in this study shared genetic similarities with A/muscovy duck/Vietnam/LBM719/2014. ^16^ The material for the investigation originated from the LBM, indicating the possibility of viral mixing between different species in the LBM (live bird market).

The specimens utilized in this study are closely linked to previously reported isolates from Indonesia, notably Yogyakarta and Central Java, ^16^ Sulawesi, ^43^ Banten and North Sumatra, ^16^ based on the phylogenetic tree. Furthermore, the isolates are similar to those found in China and Vietnam. Wild birds serve as natural reservoirs for all AIV subtypes and play a crucial role in the virus’s ecology and spread. The global spread of the AI virus is assisted by the trade in chicken and poultry products, as well as seasonal bird movement. ^44^ The passage of wild birds from East Asia to Australia has a substantial impact on the spread of the H9N2 AI virus in Chinese layer farms, and this movement can result in viral transmission between different places. ^45^

The receptors employed for comparative docking in this work are the HA1 protein from the latest isolate and A/Layer/Indonesia/WestJava-04/2017. Both were obtained using 3D modeling with PHYRE2 utilizing the corresponding amino acid sequences from GenBank. The docking results in this study were chosen based on full protein binding with an X-ray density (RMSD) value identified at 2 Å.

Based on the docking results of HA12017 and HA12023 with ligand Sia 3, a lower ∆G value was obtained for the binding of HA12017-Sia3 compared to HA12023-Sia3. This prediction suggests that the A/Layer/Indonesia/WestJava-04/2017 isolate has a better affinity for Neu5Acα(2,3)-Gal than the recent isolate from 2023. The Gibbs free energy (∆G) is one of the parameters for the stability of the ligand–receptor conformation. In living organisms, metabolic reactions are evaluated based on thermodynamics, which can be either endergonic or exergonic. ^46^ Gibbs free energy represents the energy available to do work at constant temperature and pressure and is produced during exergonic reactions when metabolic processes occur. The lower the Gibbs free energy of a molecule, the more stable it is, and the reaction proceeds spontaneously. This is in line with thermodynamic equilibrium, where a more negative ∆G indicates a more spontaneous reaction or faster formation of a stable conformation. ^47^ A lower ∆G indicates a stronger complex formation between the enzyme and ligand due to the stability and strength of noncovalent interactions within the enzyme-ligand complex. This can be observed from the amount of free energy released during the formation of interactions within the enzyme-ligand complex. This observation is also supported by other parameters, such as the number of hydrogen bonds formed. ^48^

The isolate A/Layer/Indonesia/WestJava-04/2017 forms more hydrogen bonds compared to the latest isolate. Hydrogen bonds are specific and essential interactions in the process of ligand–receptor interactions. ^49^ Therefore, hydrogen bonds contribute to the affinity of a molecule toward the target protein, forming electrostatic interactions between hydrogen donors and acceptors. The analysis of hydrogen bond interactions considers a bond distance of 3.9 Å between the donor and acceptor. ^50^ However, in the docking results between HA12017 and HA12023 with ligand Sia 6, a lower ∆G value was obtained for the binding of HA12023-Sia6 compared to HA12017-Sia6. The hydrogen bond interactions observed in this case also support the notion that the latest isolate has a stronger binding affinity for Neu5Acα(2,3)-Gal than the A/Layer/Indonesia/WestJava-04/2017 isolate. The number of hydrogen bonds identified in the docking results for HA12023-Sia6 is five, while HA12017-Sia6 forms four hydrogen bonds. More hydrogen bonds indicate a stronger ligand–receptor interaction. In summary, the A/Layer/Indonesia/WestJava-04/2017 isolate forms more hydrogen bonds, indicating its higher affinity for Neu5Acα(2,3)-Gal. Conversely, the latest isolate, HA12023, shows a lower ∆G value and forms more hydrogen bonds with the ligand Sia 6, suggesting its increased strength in binding to Neu5Acα(2,3)-Gal compared to the A/Layer/Indonesia/WestJava-04/2017 isolate.

In addition to the ∆G parameter and the number of hydrogen bonds, nonbonding residue interactions between the protein and ligand, commonly known as hydrophobic interactions, also play a role in determining the stability of Neu5Acα with HA12017 and HA12023. Hydrophobic interactions are characterized by avoiding the aqueous environment and tend to cluster within the interior of the globular protein structure, minimizing interactions of nonpolar residues with water. ^51^^,^^52^ In this study, all docking results indicated the presence of a hydrophobic residue, Leu216, which is a nonpolar amino acid. Nonpolar amino acid residues tend to form chain-like clusters in the interior part of the protein. Residues involved in hydrophobic interactions are located in the interior region of the protein, contributing to the stability of the protein’s tertiary structure. This indicates that position 216 plays a crucial role in the binding interaction of the HA1 protein with Neu5Acα.

In conclusion, this study investigated the molecular characteristics of avian influenza virus subtype H9N2 in Indonesia, focusing on a farm in West Java province from 2017 to 2023. Molecular analysis revealed 11 amino acid residue differences between the recent virus isolate and the 2017 reference isolate, yet both viruses still fell within the h9.4.2.5 subclade phylogenetically. At critical AIV sites, the recent isolate exhibited a similar arrangement to the 2017 isolate, with the exception of amino acid N at position 183 in site II of the antigenic site. Furthermore, molecular docking study indicated that the HA1 protein from the 2017 virus had a preference for binding with Neu5Ac2-3Gal, while the 2023 virus displayed a tendency to predominantly bind with Neu5Ac2-6Gal, a sialic acid receptor that commonly found in mammals. These findings highlight the evolution of avian influenza virus subtype H9N2 in the region, with notable mutations and changes in receptor binding preferences. The insights are crucial for understanding the potential risks associated with the virus’s adaptability and its interactions with host species, including humans, emphasizing the importance of continued surveillance and research efforts in managing avian influenza outbreaks.

### Ethical statement

The present research adheres to the guidelines outlined in the Indonesian Law on Animal Health Research (UU/18/2009, article 80). Due to the absence of live animals in this study, ethical approval was not necessary.

## Data Availability

GenBank: The Nucleotide database. Accession number MG957203; https://www.ncbi.nlm.nih.gov/nuccore/MG957203. ^16^ The ligands used in the simulation were obtained from https://pubchem.ncbi.nlm.nih.gov/. They are Neu5Ac2-3Gal (compound CID: 13832708) and Neu5Ac2-6Gal (compound CID: 53262334).
