# Analyzing Molecular Traits of H9N2 Avian Influenza Virus Isolated from a Same Poultry Farm in West Java Province, Indonesia, in 2017 and 2023

**DOI:** 10.12688/f1000research.150975.3

**Published:** 2025-03-05

**Authors:** Muhammad Ade Putra, Amin Soebandrio, I Wayan Teguh Wibawan, Christian Marco Hadi Nugroho Nugroho, Ryan Septa Kurnia, Otto Sahat Martua Silaen, Rifky Rizkiantino, Agustin Indrawati, Okti Nadia Poetri, Desak Gede Budi Krisnamurti

**Affiliations:** 1 Master of Animal Biomedical Sciences, School of Veterinary and Biomedical, IPB University, Bogor, West Java, 16680, Indonesia; 2 Department of Microbiology, Faculty of Medicine, University of Indonesia, Jakarta, Jakarta, 10320, Indonesia; 3 Division of Medical Microbiology, School of Veterinary Medicine and Biomedical Sciences, IPB University, Bogor, West Java, 16680, Indonesia; 4 Animal Health Diagnostic Unit, PT. Medika Satwa Laboratoris, Bogor, West Java, 16166, Indonesia; 5 Division of Central Laboratory and Disease Research Center, Technology and Research Development, Central Proteina Prima (CP Prima) Inc., Tangerang, Banten, 15560, Indonesia; 6 Department of Medical Pharmacy, Faculty of Medicine, University of Indonesia, Jakarta, Jakarta, 10430, Indonesia

**Keywords:** avian influenza, characterization, docking, H9N2, Indonesia, Mutation

## Abstract

**Background:**

Indonesia is one of the countries that is endemic to avian influenza virus subtype H9N2. This study aims to compare the molecular characteristics of avian influenza virus (AIV) subtype H9N2 from West Java.

**Methods:**

Specific pathogen-free (SPF) embryonated chicken eggs were used to inoculate samples. RNA extraction and RT–qPCR confirmed the presence of H9 and N2 genes in the samples. RT–PCR was employed to amplify the H9N2-positive sample. Nucleotide sequences were obtained through Sanger sequencing and analyzed using MEGA 7. Homology comparison and phylogenetic tree analysis, utilizing the neighbor-joining tree method, assessed the recent isolate’s similarity to reference isolates from GenBank. Molecular docking analysis was performed on the HA1 protein of the recent isolate and the A/Layer/Indonesia/WestJava-04/2017 isolate, comparing their interactions with the sialic acids Neu5Ac2-3Gal and Neu5Ac2-6Gal.

**Results:**

RT–qPCR confirmed the isolate samples as AIV subtype H9N2. The recent virus exhibited 11 amino acid residue differences compared to the A/Layer/Indonesia/WestJava-04/2017 isolate. Phylogenetically, the recent virus remains within the h9.4.2.5 subclade. Notably, at antigenic site II, the recent isolate featured an amino acid N at position 183, unlike A/Layer/Indonesia/WestJava-04/2017. Molecular docking analysis revealed a preference of HA1 from the 2017 virus for Neu5Ac2-3Gal, while the 2023 virus displayed a tendency to predominantly bind with Neu5Ac2-6Gal.

**Conclusion:**

In summary, the recent isolate displayed multiple mutations and a strong affinity for Neu5Ac2-6Gal, commonly found in mammals.

## Introduction

Avian influenza is an acute viral infectious disease that can affect all types of birds of any age. Based on the differences in hemagglutinin (H1–18) and neuraminidase (N1–N11) components, avian influenza is divided into several subtypes. ^1^^,^^2^ Each subtype has varying levels of pathogenicity, ranging from low pathogenic avian influenza (LPAI) to high pathogenic avian influenza (HPAI). However, both LPAI and HPAI can affect bird health. ^3^ The presence of gene mutations through antigenic drift and antigenic shift can result in a change in symptoms. Initially, LPAI may not cause significant harm, but with these mutations, it can become highly detrimental to the poultry industry. ^4^

AIV is an RNA virus that consists of eight segments, each encoding viral protein genes. On the other hand, this virus is also more prone to mutation than DNA viruses. ^5^ Diagnosis using polymerase chain reaction (PCR) and sequencing of the viral genome is necessary to determine the homology between the vaccine virus and the circulating field virus. ^6^ This is because if the circulating virus has low homology with the vaccine, it can result in the ineffectiveness of the vaccine due to the continued shedding of the virus. ^7^

In AIV, there is a crucial virus segment that needs to be characterized, which is hemagglutinin (HA), corresponding to the fourth segment of the AI genome. ^8^^,^^9^ HA functions at the early stage of infection by attaching the virus to the host receptor. Mutations in the HA gene can increase the virulence and pathogenicity of the virus. ^10^ Avian influenza infects its target host by initiating the recognition of the HA protein by cellular proteases from the target infected cell. Cellular proteases activate HA0, splitting it into two parts: HA1 and HA2. HA1 binds to Neu5Ac, while HA2 plays a role in fusion between the viral envelope and the host endosomal membrane. Without this recognition, the virus cannot infect the cell. ^11^

One subtype AIV that poses a threat to the poultry industry is the H9N2 subtype. ^12^ In countries affected by avian influenza outbreaks, such as China, the H9N2 subtype of AIV causes significant losses due to its high morbidity rate of up to 100% in infected layer chicken farms. ^13^ Infected layer chickens experience a decrease in appetite accompanied by a drastic decline in egg production. Viral infection is often exacerbated by secondary infections from bacteria or other viruses, resulting in high mortality among the infected layer chicken population. ^14^ H9N2 virus infection may not be immediately visible at onset, but the virus spreads rapidly through the shedding of feces or nasal discharge from birds. Due to these characteristics, rapid and accurate detection is crucial to identify the presence of AIV infection on a farm, enabling appropriate measures to be taken in addressing H9N2 virus infection. ^15^

West Java Province is a province in Indonesia that has several densely populated districts for layer chicken farming, including Bogor, Sukabumi, Cianjur, Ciamis, and several other districts. In 2017, one H9N2 isolate was successfully isolated and characterized, namely, strain A/Layer/Indonesia/WestJava-04/2017 (H9N2) (GenBank accession number MG957203), originating from a densely populated layer chicken farming area in West Java. ^16^ The high density of layer chicken farms in a region facilitates the spread and mutation of AIVs. Therefore, this study aims to isolate and characterize the H9N2 subtype AIV from the layer chicken farm where the A/Layer/Indonesia/WestJava-04/2017 (H9N2) strain originated. This study also describes the molecular changes in the HA gene using molecular docking after implementing the H9N2 vaccination program, which has been ongoing for six years.

## Methods

### Sample collection

Sampling was conducted at a commercial layer chicken farm in West Java Province, the original location of the AIV subtype H9N2 strain A/Layer/Indonesia/WestJava-04/2017 (H9N2) (GenBank Accession No. MG957203). After five years of vaccination with a homologous commercial vaccine at the farm, a decrease in production and bird mortality occurred in February 2023. Samples consisting of brain, trachea, and oviduct organs from three deceased chickens with symptomatic of H9N2 infection. The samples were from flock of 100,000 chickens at a commercial egg production farm. All organs from all chickens combined in one sample and then was tested by virus isolation and PCR methods.

### Virus isolation

The sample was multiplied using standard laboratory procedures. Tissue of pooled organs of three chickens was combined with sterile phosphate-buffered saline (pH 7.4) from ThermoFisher (28348) containing antibiotics (200 g/ml penicillin and 100 g/ml streptomycin) and then centrifuged at 1,000 g and 4 °C for fifteen minutes. The remaining supernatant was filtered through a 0.45 μm membrane filter and injected intra-allantoically into 9-day-old SPF eggs. The SPF eggs were incubated at 37 °C for 48–72 hours, and daily monitoring of embryo mortality was performed. ^17^ After 72 hours of incubation, all ECEs, regardless of their viability, were subsequently preserved at a refrigerated temperature of 8 °C overnight and then harvested. The collected specimens consisted of allantoic fluid, which was subsequently utilized for conducting a rapid HA assay to promptly detect AIV in early stages. ^16^

### Viral RNA was identified using a reverse transcription-polymerase chain reaction (RT–PCR) assay

The RNA was further extracted from the allantoic fluid that was collected using a total RNA mini kit reagent from Geneaid ^®^, Taiwan (RPD050). The RNA obtained was then analyzed for the presence of the H9N2 virus using the SensiFASTTM SYBR Lo-ROX Kit from Bioline ^®^, Taunton (BIO-94005). For the RT–qPCR assay, the primers H9-F: 5′-ATCGGCTGTTAATGGAATGTGTT-3′, H9-R: 5′- TGGGCGTCTTGAATAGGGTAA-3′ ^18^ and N2-F: 5′-CTCCAATAGACCCGTACTAT-3′, N2-R: 5′-CCTGAAGTCCCACAAAATAC-3′ ^19^ were utilized with the LongGene Q2000C (China). The thermal profile for gene amplification included two minutes of polymerase activation at 95 °C, which was followed by a total of 45 cycles of denaturation at 95 °C for 5 seconds, annealing at 60 °C for 10 seconds, and extension at 72 °C for 15 seconds. When the cycle threshold (Ct) value falls below 40, a positive result is indicated. In addition, RT–PCR assays were conducted to detect avian influenza subtype H9N2, Newcastle disease virus (NDV), and infectious bronchitis virus (IBV). RT–PCR was conducted according to the protocol used in a previous study. ^20^

### Sequencing

The confirmed HA of the H9 gene from RT–PCR was amplified for sequencing using the MyTaq One-Step RT–PCR kit from Bioline ^®^, Taunton (BIO-65049), and the primers HAp1-F: 5′-TCCACGGAAACTGTAGACACA-3′, HAp1-R: 5′-TTCTGTGGCTCTCTCCTGAAA-3′ and Hap2-F: 5′-AGGCCTCTTGTCAACGGTTT-3′, Hap2-R: 5′-CCAACGCCCTCTTCACTTTA-3′ were used. ^21^ The Sanger sequencing method was performed by First BASE Laboratories, Malaysia, which separated the PCR products using electrophoresis and purified the desired band for sequencing. Using Bioedit v.7 ( https://bioedit.software.informer.com/7.0/), the nucleotide and amino acid sequences of a recently isolated strain’s HA gene were determined, and ClustalW was used for alignment. A modern virus phylogenetic tree was constructed using MEGA v 7.0 ( https://www.megasoftware.net/), the neighbor-joining method, and 1,000 alignment repetitions. The phylogenetic tree was compared the clade of the recent study isolate, a previous isolate A/Layer/Indonesia/WestJava-04/2017 (H9N2) (GenBank Accession No. MG957203) and other prototype H9N2 isolate strains of the all H9N2 clades from GenBank. The genetic distance between isolates and the topology of the phylogenetic tree were used to compared strains. The genetic distance between isolates and the topology of the phylogenetic tree were used to compared strains. The genetic similarity percentage of all study strains was calculated by using the maximum composite likelihood model implemented in MEGA v 7.0 ( https://www.megasoftware.net/). ^22^

Several key regions were investigated in this investigation, including the receptor-binding sites (RBS) on the left and right edges of the binding pocket, as well as the cleavage site. In addition, the correlation between particular amino acid residues (54, 80, 106, 109, 113, 123, 125, 129, 130, 135, 137, 146, 147, 149, 150, 152, 164, 165, 178, 179, 182, 183, 188, 189, 194, and 216) and H9N2 virus antigenicity was analysed. The amino acid sequence of the HA gene from the most recent H9N2 virus was uploaded to http://www.cbs.dtu.dk/services/NetNGlyc/to evaluate potential N-glycosylation sites. ^21^

### Molecular Docking of Protein HA1 (2017 and 2023 isolates) with Ligands Neu5Ac2-3Gal and Neu5Ac2-6Gal

The simulation was conducted on a computer hardware system with the following specifications: 8.00 GB RAM and an Intel ^®^ Core i5-2520 M CPU with a clock speed of 2.50 GHz. The software used in the study included Google Chrome v114.0.5735.199 for accessing the SwissDock website, which served as the protein–ligand docking server and ran automatically ( http://swissdock.ch/docking). Additionally, BIOVIA Discovery Studio Visualizer v21.1.0.20298 ( https://discover.3ds.com), Chimera v1.17.3 ( https://www.cgl.ucsf.edu/chimera/download.html), AutoDock Tools v1.5.7 ( https://autodock.scripps.edu/), PyMol v2.5.2 ( https://pymol.org/), and LigPlot+ v2.2.8 ( https://www.ebi.ac.uk/thornton-srv/software/LigPlus/download.html) were used for data analysis and visualization.

The simulation begins with the creation of a 3D model of the HA1 protein from the AIV, specifically the recent study isolate (HA1-2023) and the A/Layer/Indonesia/WestJava-04/2017 strain (HA1-2017). This is achieved using the PHYRE2 Protein Fold Recognition Server ( http://www.sbg.bio.ic.ac.uk/). The ligands used in the simulation were obtained from https://pubchem.ncbi.nlm.nih.gov/. They are Neu5Ac2-3Gal (compound CID: 13832708) and Neu5Ac2-6Gal (compound CID: 53262334).

The docking was performed using the automated server facility on SwissDock, where the uploaded documents were in PDB format (.pdb) for the protein acting as the receptor and SYBYL MOL2 format (.mol2) for the molecules acting as ligands. Docking was carried out for the protein HA1-2017 with the Neu5Ac2-3Gal ligand, HA1-2017 with the Neu5Ac2-6Gal ligand, HA1-2023 with the Neu5Ac2-3Gal ligand, and HA1-2023 with the Neu5Ac2-6Gal ligand. Only models with ligands attached to the Leu216 residue region are observed and selected for further analysis.

The selection of the residue is based on the fact that the amino acid at position 216 determines the virus’s tendency to infect mammalian cells dominated by Neu5Ac2-6Gal or avian cells dominated by Neu5Ac2-3Gal. ^20^ The docking results were downloaded in the Chimera Web Data format (.xml) and run in Chimera software v1.17.3 to select the best docking model. The best model was chosen based on the Gibbs free energy (ΔG) value, where ΔG represents the stability parameter of the bond formed between the protein and the ligand. A more negative value indicates a more stable bond. The protein–ligand interactions are then evaluated, observing hydrogen bonding, hydrogen bond distances (Å), interacting residues and functional groups, and residues that interact with the ligand noncovalently. ^23^

### Nucleotide sequence accession numbers

The partial CDS of the HA gene of A/chicken/Indonesia/MSL0123/2023 (H9N2) in this study was deposited in GenBank and received the accession number OR243721.

## Results

### H9N2 subtype AI virus detection

The presence of viral RNA was identified by RT–qPCR specifically targeting H9 and N2. The results shown in Supplementary Figure 1a and Supplementary Figure 1b indicate that the recent isolate of this study is an AIV subtype H9N2. The H9 gene in a recent isolate was amplified with a Ct value of 28.50, while the N2 gene was amplified at a Ct value of 34.69.

### Amplification of the Hemagglutinin (HA) gene

The results of HA gene amplification using primers HApar1 and HApar2 on a recent sample from this investigation are shown in Supplementary Figure 1c. The electrophoresis results revealed the presence of DNA bands at the 736 bp (for HApar1) and 712 bp (for HApar2) sites. Marker 100-2000 bp is the size of the molecular marker displayed in the illustration.

### Amino acid analysis

The partial CDS of the HA gene from this study was deposited in GenBank, with the accession number OR243721 as A/chicken/Indonesia/MSL0123/2023 (H9N2). The amino acid sequence at the cleavage site of the recent isolate is PSRSSR↓GLF, while the receptor binding site has the motif PWTNTLY ( Table 1). Additionally, at position 217 on the left side of the receptor binding site (RBS), the recent isolate contained the amino acid M (methionine). It should be noted that this amino acid sequence is identical to A/Layer/Indonesia/WestJava-04/2017, which originated from the same farm.

**Table 1.** Amino acid analysis of important sites in the HA gene of H9N2 viruses.

A. Receptor-binding pockets, cleavage sites and antigenic site of recent isolate of H9N2 compared to A/Layer/Indonesia/WestJava-04/2017 (H9N2) *^a^*								
Virus	Lineage	Receptor binding site	Left-edge of binding pocket	Right-edge of binding pocket	Cleavage site	Antigenic site		Acession Number
						Site I	Site II	
A/Layer/Indonesia/WestJava-04/2017 (H9N2)	h9.4.2.5	PWTNTLY	NGLMGR	GTSKA	PSRSSR↓GLF	SKP	DDL	MG957203
A/chicken/Indonesia/MSL0123/2023 (H9N2)	h9.4.2.5	PWTNTLY	NGLMGR	GTSKA	PSRSSR↓GLF	SKP	DNL	OR243721

B. Potential N-glycosylation sites _2)_									
					HA1				
Virus	11–13 ^b^	123–125	127–202	178–180	188–190	200–202	280–282	287–289	295–297
A/Layer/Indonesia/WestJava-04/2017 (H9N2)	NST	NVS	–	–	–	NRT	NTT	NVS	NCS
A/chicken/Indonesia/MSL0123/2023 (H9N2)	NST	NVS	–	–	–	NRT	NTT	NVS	NCS

C Other mutations of recent isolate										
					HA1					
Virus	23	34	53	69	72	74	114	120	163	179
A/Layer/Indonesia/WestJava-04/2017 (H9N2)	N	H	D	P	E	R	I	K	E	T
A/chicken/Indonesia/MSL0123/2023 (H9N2)	D	Q	E	S	G	K	T	T	G	D

^a^ The receptor-binding site (RBS) includes residues at positions 92, 143, 145, 173, 180, 184, and 185. The left-edge of the binding pocket is located at positions 214-219, while the right-edge of the binding pocket is situated at positions 128-132. The cleavage site is positioned at positions 315-323. The antigenic site is divided into two regions: site I, which includes positions 125, 147, and 152, and site II, which encompasses positions 135, 183, and 216.

^b^ The arrangement of amino acid residues in the HA genes of AIV subtype H9N2.These residue placements are based on the H9 numbering system.

In a recent study, the antigenic site I of the HA gene in the recent sample displayed the same motif as A/Layer/Indonesia/WestJava-04/2017 (H9N2). At site I, amino acids S (serine), K (lysine), and P (proline) were present. For site II, the motif consisted of amino acids D (aspartate), N (asparagine), and L (leucine) at positions 135, 183, and 216. In contrast, A/Layer/Indonesia/WestJava-04/2017 (H9N2) exhibited D (aspartate) at position 183. The analysis of the HA gene in the recent sample included an examination of potential glycosylation sites (PGS). The HA1 gene segment revealed the NXT/S motif (where X represents any amino acid except proline) at positions 11-13 (NST), 123-125 (NVS), 200-202 (NRT), 280-282 (NTT), 287-289 (NVS), and 295-297 (NCS). There were also several other amino acid differences observed at positions 23, 34, 53, 69, 72, 74, 114, 120, 163, and 179 ( Table 1).

### Homology comparison

The results of the homology comparison are shown in Table 2. The recent isolate has a similarity of 95.84% with A/Layer/Indonesia/WestJava-04/2017 (H9N2), which was isolated from the same farm but in a different year. When analyzed against several representatives of the H9N2 subtype clades, the recent isolate has a similarity of approximately 72.24% with clade h9.1, 72.56% with clade h9.2, 74.72% with clade h9.3, and 81.80% with h9.4.1. These results are lower compared to the similarity of the recent isolate with viruses classified under clade h9.4.2. Based on homology comparison, a recent isolate, A/chicken/Indonesia/MSL0123/2023 (H9N2), belonged to subclade h9.4.2.5.

**Table 2.** Nucleotide sequence similarities between the current strain and several H9N2 isolates in GenBank.

No.	Strain	Accession No.	Clade	Similarity (%)
1	A/turkey/California/189/66 (H9N2)	AF156390	h9.1	72.24
2	A/Shorebird/Delaware/9/96 (H9N2)	AF156386	h9.2	72.56
3	A/Duck/Hong_Kong/Y439/97 (H9N2)	AF156377	h9.3	74.72
4	A/Quail/Hong_Kong/G1/97 (H9N2)	AF156378	h9.4.1	81.80
5	A/Chicken/Shanghai/F/98 (H9N2)	AY743216	h9.4.2.1	86.05
6	A/guineafowl/HongKong/NT184/03 (H9N2)	AY664674	h9.4.2.2	83.66
7	A/Chicken/Guangdong/SS/94 (H9N2)	AF384557	h9.4.2.3	86.95
8	A/Duck/Hong_Kong/Y280/97 (H9N2)	AF156376	h9.4.2.4	88.30
9	A/chicken/Henan/LY-36/2013 (H9N2)	KF638574	h9.4.2.5	90.95
10	A/Layer/Indonesia/WestJava-04/2017 (H9N2)	MG957203	h9.4.2.5	95.84
11	A/muscovy_duck/Vietnam/LBM719/2014 (H9N2)	LC028176	h9.4.2.5	95.55
12	A/chicken/Guangdong/FZH/2011 (H9N2)	JF715024	h9.4.2.6	84.21

### Phylogenetic tree

**Figure 1.**
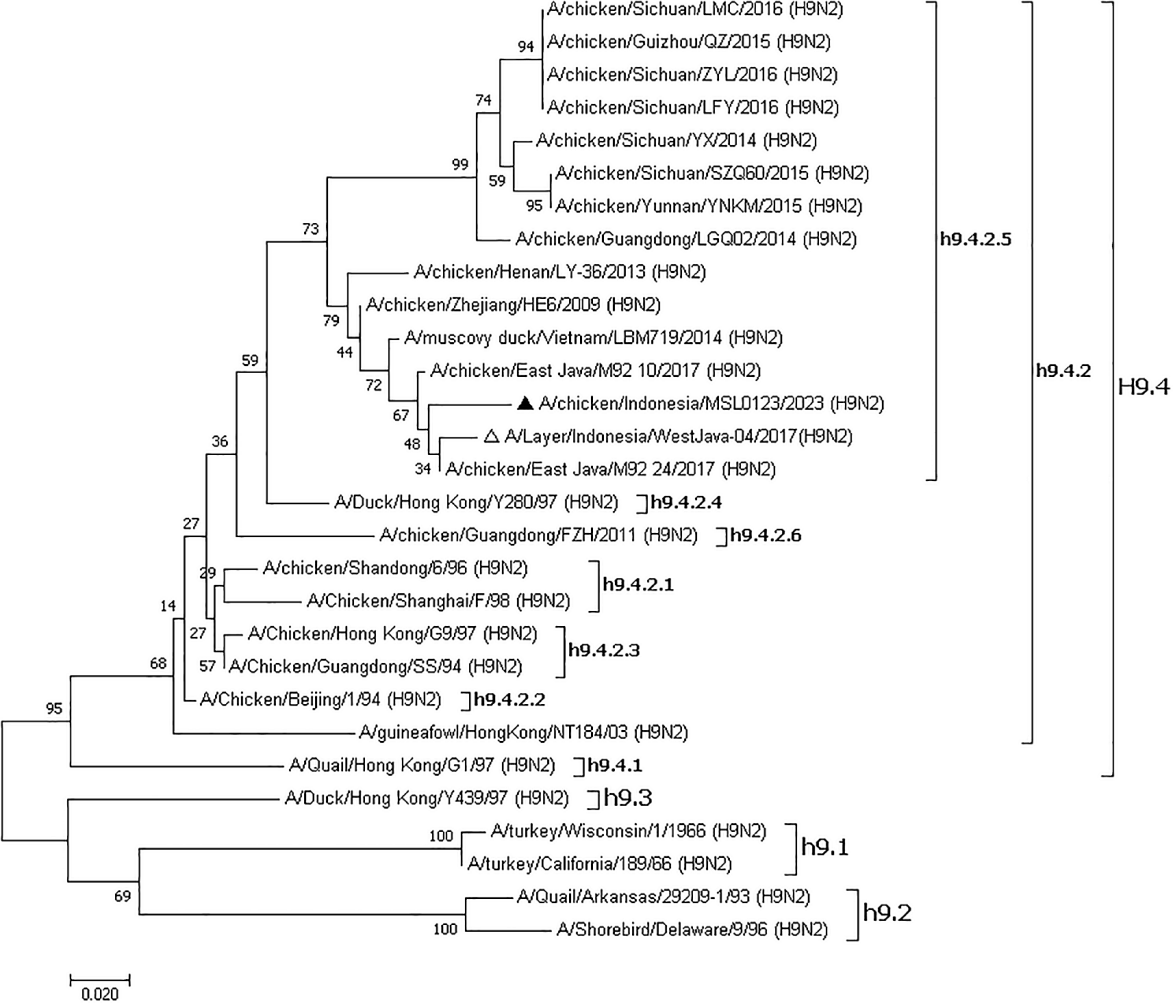
Phylogenetic tree of the hemagglutinin (HA) gene of the AIV H9N2 virus was constructed.

The phylogenetic tree showed that the samples were together with other isolates from Indonesia such as A/Layer/Indonesia/WestJava-04/2017 (H9N2), A/chicken/East_Java/M92_10/2017 (H9N2), A/chicken/East_Java/M92_24/2017 (H9N2), Vietnam A/muscovy_duck/Vietnam/LBM719/2014 (H9N2) and China such as A/chicken/Zhejiang/HE6/2009 (H9N2), A/chicken/Henan/LY-36/2013 (H9N2), A/chicken/Guangdong/LGQ02/2014 (H9N2) so that these were included in China, Vietnam, and Indonesia (CVI) clades ( Figure 1). Recent viruses belonged to subclade h9.4.2.5, according to a phylogenetic study.

### Docking analysis

**Figure 2.**
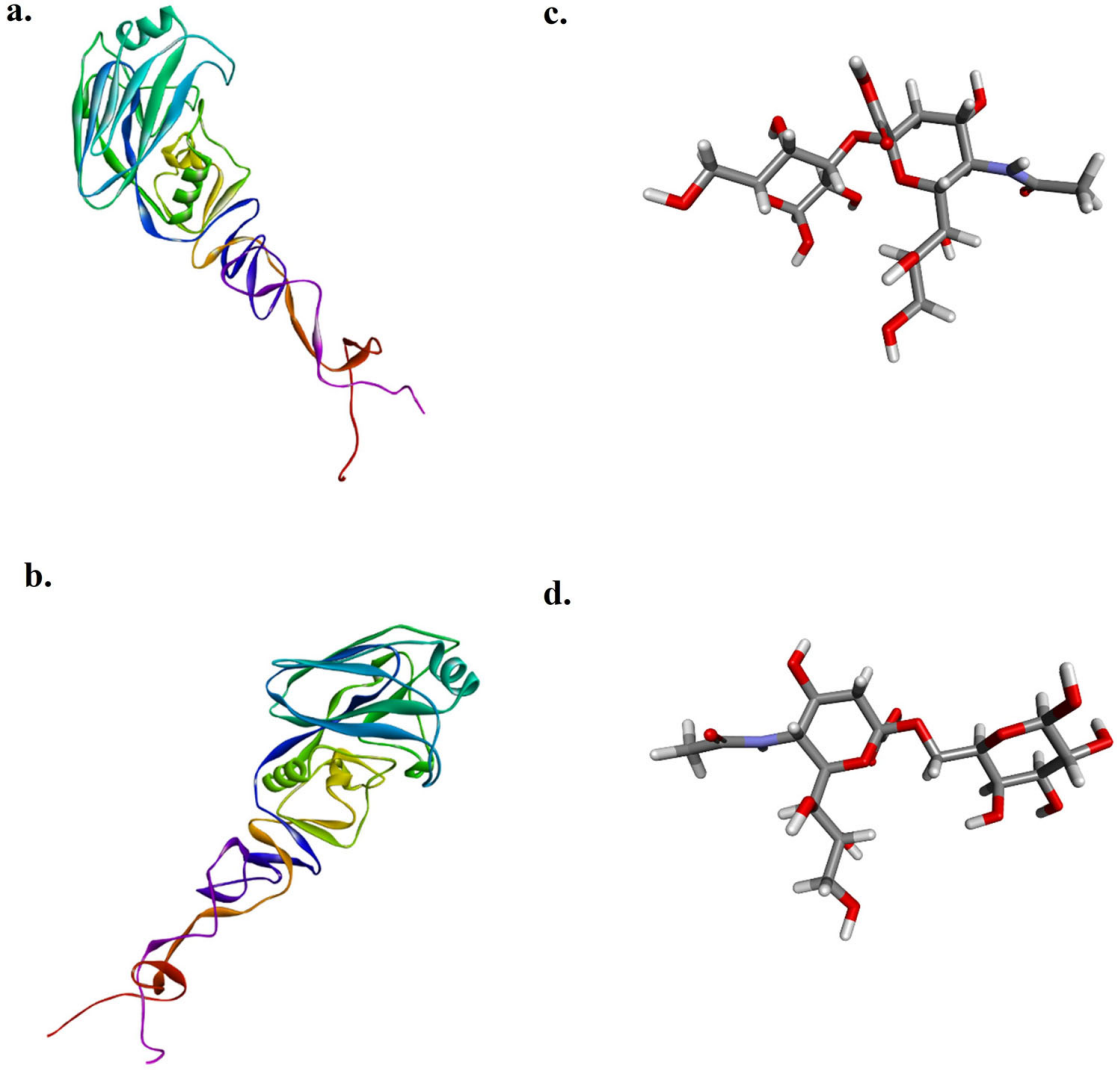
Modeled protein HA1 of H9N2 predicted by the Robetta server, visualized in Discovery Studio Visualizer.

The target HA1 proteins of AIV subtype H9N2 isolated in 2017 (HA12017) and 2023 (HA12023) were chosen for the study because of their importance in viral attachment for the docking analysis, as there were no 3D structures of the protein, and its modeling was carried out. After submission of their amino acid sequences on Phyre2, the modeled proteins obtained were downloaded and visualized in Discovery Studio Visualizer. The Neu5Ac2-3Gal (Sia3) and Neu5Ac2-6Gal (Sia6) ligands used in this study are shown in Figure 2.

**Figure 3.**
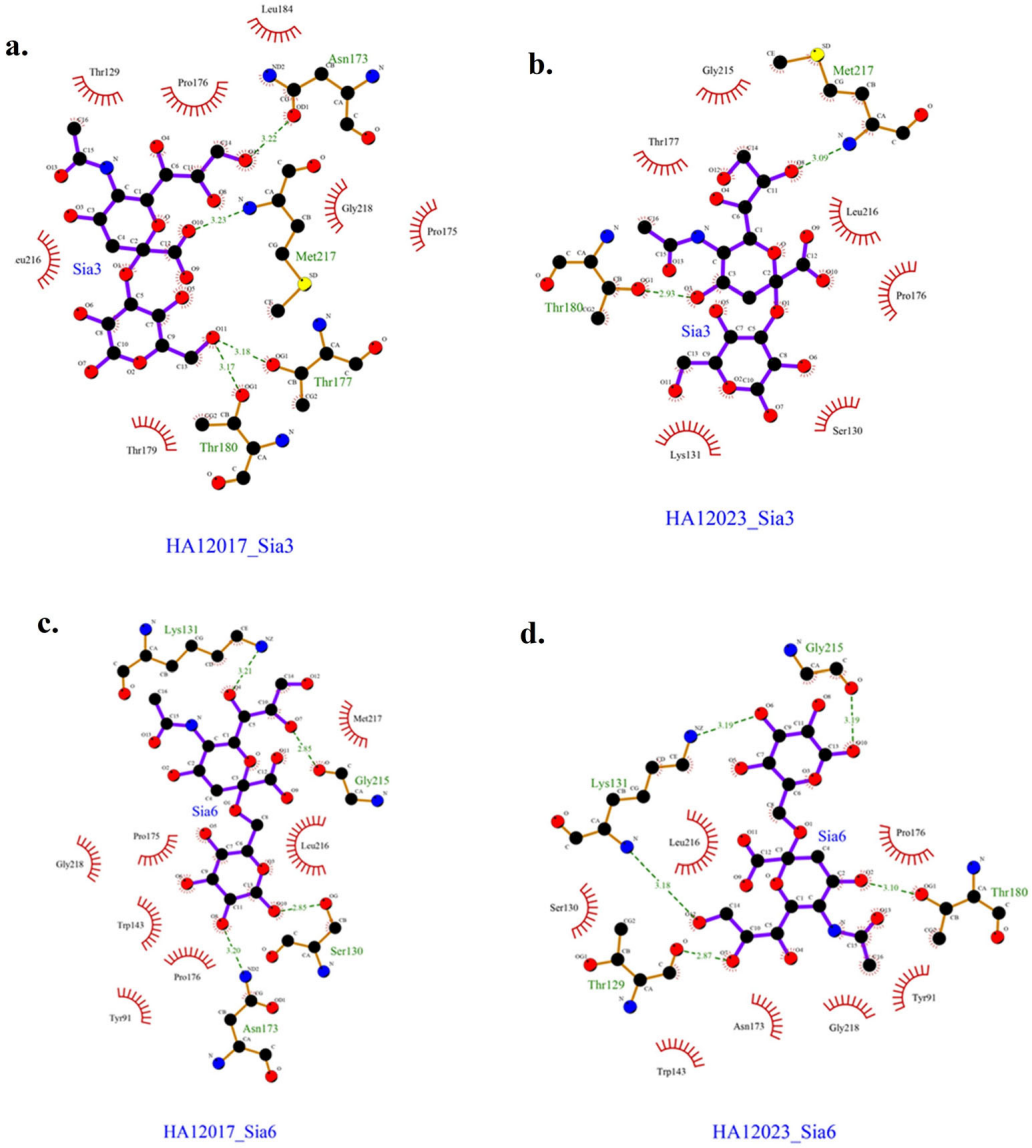
Interactions occurring in HA12017 and HA12023 with ligand Sia3 and Sia6.

Based on the docking results, the data obtained are presented in Table 3. The obtained results represent the best model with the most negative ∆G and the ligand positioned and bound near the Leu216 residue. The interactions between each protein and ligand are shown in Figure 3, with hydrogen bonding data and residues involved in hydrogen bonding interactions presented in Table 4. The interactions that occur can be either hydrogen bonding or nonbonding interactions that strengthen the ligand’s affinity with the protein.

**Table 3.** Docking results of proteins HA12017 and HA12023 with ligands Sia3 and Sia6 selected.

Protein	Ligand	Cluster	Cluster rank	Delta G ( ∆ G) (Kcal/mol)
HA12017	Sia3	2	0	-7.98304
HA12017	Sia6	11	0	-7.79904
HA12023	Sia3	2	0	-7.42221
HA12023	Sia6	8	0	-7.84408

**Table 4.** Hydrogen bonds and interacting residues.

Protein	Ligand	Hydrogen bond distance (Å)	Residues and functional groups that are binding	Residues that have nonbonding interactions with the ligand
HA12017	Sia3	3.22	Asn173 (O-O)	Thr129, Pro178, Leu184, Pro175, Gly218, Thr179, Leu216
		3.23	Met217 (O-N)	
		3.18	Thr177 (O-O)	
		3.17	Thr180 (O-O)	
HA12023	Sia3	2.93	Thr180 (O-O)	Thr177, Gly215, Leu216, Pro176, Ser130, Lys131
		3.09	Met217 (O-N)	
HA12017	Sia6	3.21	Lys131 (O-N)	Gly218, Pro175, Trp143, Pro176, Tyr91, Leu216, Met217
		2.85	Gly215 (O-O)	
		2.85	Ser130 (O-O)	
		3.20	Asn173 (O-N)	
HA12023	Sia6	3.18	Lys 131 (O-N)	Ser130, Leu216, Pro176, Tyr91, Gly218, Asn173, Trp143
		3.19	Lys 131 (O-N)	
		3.19	Gly215 (O-O)	
		3.10	Thr180 (O-O)	
		2.87	Thr129 (O-O)	

## Discussion

This study provides a comprehensive molecular comparison of the H9N2 avian influenza virus (AIV) isolates from 2017 and 2023 from the same poultry farm in West Java, Indonesia. The findings highlight key genetic mutations, antigenic site variations, and receptor-binding preferences of HA that may influence viral evolution and host adaptation. ^24^^,^^25^

Phylogenetic analysis confirms that both isolates belong to the h9.4.2.5 subclade, indicating evolutionary stability within this lineage despite ongoing vaccination efforts. The results suggest that while genetic changes occur over time, the virus remains within a well-defined evolutionary framework, allowing for targeted monitoring and control strategies. The specimens utilized in this study are also closely linked to previously reported isolates from Indonesia, notably Yogyakarta and Central Java, ^16^ Sulawesi, ^26^ Banten, and North Sumatra, ^16^ based on the phylogenetic tree. Wild birds serve as natural reservoirs for all AIV subtypes and play a crucial role in the virus's ecology and spread. The trade assists the global spread of the AI virus in chicken and poultry products and seasonal bird movement. ^27^ The passage of wild birds from East Asia to Australia substantially impacts the spread of the H9N2 AI virus in Chinese layer farms, and this movement can result in viral transmission between different places. ^28^

Molecular analysis reveals 11 amino acid substitutions between the 2017 and 2023 isolates, suggesting ongoing genetic drift within the HA gene. The substitution at antigenic site II is significant, where the 2023 isolate features an N183 residue instead of D183 in the 2017 strain. This mutation is known to influence antigenicity and immune recognition, potentially affecting vaccine efficacy. ^29^ In vivo studies demonstrated that changes influenced viral replication and transmission of H9N2 in chickens. D183-containing viruses were able to multiply in the lungs of infected hens. Variations in antigenic site II locations 183 and 216 decrease viral interactions with epitope-specific antibodies and can result in mutant virus escape. ^16^^,^^30^ The ability of the virus to undergo antigenic variation is a crucial factor in its persistence and continued circulation in poultry populations, underscoring the need for periodic vaccine updates to match emerging variants.

In addition to antigenic site variations, the receptor-binding properties of the virus have also evolved. Molecular docking analysis demonstrates a shift in receptor-binding preference, with the HA1 protein of the 2017 virus exhibiting a stronger affinity for Neu5Ac2-3Gal, a receptor predominantly found in avian hosts. In contrast, the 2023 isolate shows increased binding affinity for Neu5Ac2-6Gal, a sialic acid receptor commonly found in mammals. This shift could indicate an increased potential for cross-species transmission, raising concerns about zoonotic risks. The mutation at position 216 within the antigenic region plays a key role in this altered binding affinity, reinforcing the need for enhanced surveillance of circulating H9N2 strains. ^31^

This study provided a comprehensive molecular characterization of the H9N2 avian influenza virus (AIV) isolates from 2017 and 2023, a significant limitation is the exclusive focus on the hemagglutinin (HA) gene. The HA is a critical determinant of antigenicity and host receptor binding. However, adapting AIV to vaccination and host immune responses involves multiple viral segments beyond HA. Neuraminidase (NA) is essential in viral release and spread, influencing antigenicity and immune evasion. Mutations in the NA gene can enhance viral fitness, particularly in response to selective pressures from vaccination. ^32^ Beyond HA and NA, the internal genes of the influenza virus, including polymerase genes (PB2, PB1, and PA), nucleoprotein (NP), matrix (M1 and M2), and non-structural proteins (NS1 and NS2) significantly impact viral adaptation. ^33^

The ability of the 2023 strain to favor Neu5Ac2-6Gal receptors suggests that the virus may be adapting to recognize mammalian-like receptors more efficiently. ^34^ This adaptation could lead to host range and transmission dynamics changes, warranting further investigation into potential zoonotic threats. Understanding these receptor-binding changes is crucial for assessing public health risks and developing preventive measures. In this study, the binding ability to sialic acids of the 2017 virus and 2023 virus was analyzed through molecular docking instead of direct experiment data. Conducting a receptor binding assay, such as using analogs of different sialic acids, will be more convincing in the next study. Additionally, further experimental validation, such as antigenic cartography, in vivo assays, and serological cross-reactivity studies, would be necessary to confirm the functional impact of mutations in these viral segments. Expanding surveillance efforts beyond HA sequencing would provide a more comprehensive understanding of viral evolution and vaccine-driven selection pressures in endemic regions such as Indonesia.

A limitation of this study is its reliance on samples from only one farm. While the virus's molecular characterization provides valuable insights, analyzing the adaptation potential of H9N2 strains to vaccination requires a broader sampling strategy that includes multiple farms from the region. Sampling from diverse farms would help capture genetic variation across different poultry populations and improve the generalizability of the findings.

These findings emphasize the importance of continuously monitoring circulating H9N2 strains, particularly in regions with intensive poultry farming. Surveillance programs should identify antigenic drift, evaluate receptor-binding changes, and assess vaccine effectiveness against emerging variants. Additionally, further studies incorporating in vivo receptor-binding assays and serological analysis will provide deeper insights into the implications of these molecular changes for poultry and public health.

Overall, the evolution of H9N2 in Indonesia reflects the complex interplay between viral adaptation, immune selection pressure, and potential cross-species transmission. Maintaining vigilance in genomic surveillance and vaccine updates is essential to mitigate the risks of this evolving virus and safeguard animal and human health.

### Ethical statement

The present research adheres to the guidelines outlined in the Indonesian Law on Animal Health Research (UU/18/2009, article 80). Due to the absence of live animals in this study, ethical approval was not necessary.

## Data Availability

GenBank: The Nucleotide database. Accession number MG957203; https://www.ncbi.nlm.nih.gov/nuccore/MG957203. ^16^ The ligands used in the simulation were obtained from https://pubchem.ncbi.nlm.nih.gov/. They are Neu5Ac2-3Gal (compound CID: 13832708) and Neu5Ac2-6Gal (compound CID: 53262334). Zenodo: Supplementary Figure 1, https://doi.org/10.5281/zenodo.14934564. ^35^ Data are available under the terms of the Creative Commons Attribution 4.0 International license (CC-BY 4.0).
