# Supplementary material for: Analyzing Molecular Traits of H9N2 Avian Influenza Virus Isolated from a Same Poultry Farm in West Java Province, Indonesia, in 2017 and 2023
Source: F1000Res. 2025 Mar 5;13:571. Originally published 2024 Jun 4. [Version 3] doi: 10.12688/f1000research.150975.3 (PMC11602698; doi:10.12688/f1000research.150975.3)
Supplement: Peer review history

# Reviewer report: Reviewer response for version 2

**DOI:** 10.5256/f1000research.171635.r333988

**Published:** 2024-11-27

**Authors:** Albert Perlas

**Affiliations:** 1 Helmholtz AI, Helmholtz Zentrum München, Helmholtz AI, 85764, Spain; 2 Helmholtz Pioneer Campus, Helmholtz Zentrum Muenchen, Neuherberg, 85764, Germany

The authors compared the molecular characteristics of a subtype H9N2 avian influenza virus strain isolated in 2023 with another strain isolated in 2017 from the same farm in Indonesia. They sequenced the HA segment of the 2023 strain after RT-PCR amplification using Sanger sequencing. Based on these sequencing results, the authors performed phylogenetic analysis, molecular homology comparisons, and molecular docking analysis. The results revealed 11 amino acid residue differences between the two strains, confirmed that both strains belong to the H9.4.2.5 subclade, and suggested, through molecular docking, a preference of the 2023 strain for Neu5AC2-6Gal, which is more commonly found in mammalian species. Overall, the manuscript provides relevant information on the concerning H9N2 avian influenza subtype. However, the text is not consistently clear or well-organized, and improvements in these areas would enhance the quality of the work. 

Comments

- The authors should clarify the criteria used to include other H9N2 strains in the phylogenetic analysis, aside from the two isolates from the same farm in West Java

 

- I agree with Reviewer 1 that the authors should refer to the 'recent strain' by its correct influenza strain name, adhering to the proper nomenclature.

 

- Figure 1 could be moved to the supplementary material as it does not add critical information to the manuscript.

 

- Additionally, the authors should clarify how the percentage of similarity between strains, as shown in Table 2, was calculated. 

 

- The discussion should address the potential role of other viral segments, beyond HA, in the adaptation of avian influenza to vaccination. The focus solely on HA sequencing represents a significant limitation of this work, which should be clearly acknowledged.

 

- It would be valuable to discuss the role of low-frequency variants in adaptation to vaccination, not just the consensus sequence data. This aspect is missing due to the sequencing technique employed and should be highlighted as a limitation.

 

- Another major limitation of the study is that it relies on samples from only one farm. To effectively analyze the adaptation potential of H9N2 strains to vaccination, a broader sampling strategy that includes multiple farms from the region would be necessary. If expanding the sample set is not feasible, this limitation should be explicitly discussed.

 

- The discussion section is currently written in a somewhat confusing manner. Some content belongs in the results section, while other parts would be more appropriate in the introduction. The discussion should be rewritten to reduce its length and focus on the main findings of the manuscript:
  - Both strains belong to the same clade.
  - Specific amino acid mutations, particularly in antigenic site.
  - The preference of the 2023 strain for Neu5Ac2-6Gal.

Is the work clearly and accurately presented and does it cite the current literature?

Partly

If applicable, is the statistical analysis and its interpretation appropriate?

Not applicable

Are all the source data underlying the results available to ensure full reproducibility?

Yes

Is the study design appropriate and is the work technically sound?

Yes

Are the conclusions drawn adequately supported by the results?

Yes

Are sufficient details of methods and analysis provided to allow replication by others?

Yes

Reviewer Expertise:

virology, avian influenza, genomics, animal health

I confirm that I have read this submission and believe that I have an appropriate level of expertise to confirm that it is of an acceptable scientific standard, however I have significant reservations, as outlined above.

# Reviewer report: Reviewer response for version 2

**DOI:** 10.5256/f1000research.171635.r323384

**Published:** 2024-10-09

**Authors:** Mark Zanin, Hui Lei

**Affiliations:** 1 The University of Hong Kong, Hong Kong SAR, China; 2 Centre for Immunology & Infection, Hong Kong, Hong Kong

The authors have addressed the comments raised in the review.

Is the work clearly and accurately presented and does it cite the current literature?

Yes

If applicable, is the statistical analysis and its interpretation appropriate?

Not applicable

Are all the source data underlying the results available to ensure full reproducibility?

No source data required

Is the study design appropriate and is the work technically sound?

Yes

Are the conclusions drawn adequately supported by the results?

Partly

Are sufficient details of methods and analysis provided to allow replication by others?

Yes

Reviewer Expertise:

Influenza virus zoonosis and interspecies transmission

We confirm that we have read this submission and believe that we have an appropriate level of expertise to confirm that it is of an acceptable scientific standard.

# Reviewer report: Reviewer response for version 1

**DOI:** 10.5256/f1000research.165593.r303175

**Published:** 2024-08-21

**Authors:** Mark Zanin, Hui Lei

**Affiliations:** 1 The University of Hong Kong, Hong Kong SAR, China; 2 Centre for Immunology & Infection, Hong Kong, Hong Kong

The authors conducted this study to compare the molecular characteristics of avian influenza virus subtype H9N2 isolated from West Java in 2017 and 2023. They collected samples including brain, trachea, and oviduct pooled from deceased chickens and inoculated them into specific pathogen-free embryonated chicken eggs for virus isolation. RNA extraction and RT-qPCR were used to confirm the presence of H9 and N2 genes in the samples. Sequences were obtained through RT-PCR and sanger sequencing from positive samples and the similarity of the recent isolates sequences were compared to reference isolates in Genbank through phylogenetic trees. They also compared the interactions of the recent isolates with the sialic acids Neu5Ac2-3Gal and Neu5Ac2-6Gal to that of the isolate A/Layer/Indonesia/WestJava-04/2017 (H9N2). They found that the recent virus exhibited 11 amino acid residue differences from A/Layer/Indonesia/WestJava-04/2017 (H9N2). Phylogenetically, the recent virus is also of the h9.4.2.5 subclade. The recent isolate had amino acid ‘N’ at position 183 at antigenic site II, which was different from A/Layer/Indonesia/WestJava-04/2017 (H9N2). Molecular docking analysis revealed a preference of HA1 from the 2017 virus for Neu5Ac2-3Gal, while the 2023 virus displayed a tendency to predominantly bind with Neu5Ac2-6Gal.

 These findings help to gain insights into the evolution of avian influenza virus subtype H9N2 in the region, necessary for understanding the potential risks associated with them. Overall the manuscript is well writted and well presented.

 MAJOR COMMENTS;

- No figure legends are included.
- The binding ability to sialic acids of the 2017 virus and 2023 virus were analyzed through molecular docking instead of direct experiment data. Conducting a receptor binding assay such as using analogues of different sialic acids will be more convincing. This should be noted as a limitation or a consideration in the discussion.
- Please use correct influenza strain name nomenclature, i.e., A/Layer/Indonesia/WestJava-04/2017 (H9N2).
- The authors should refer to the ‘recent isolate’ by its correct influenza strain name using correct nomenclature in the abstract and introduction after calling it the recent isolate as this is more informative.

MINOR COMMENTS;

Abstract

 “ *Homology comparison and phylogenetic tree analysis, utilizing the neighbor-joining tree method, assessed the recent isolate’s similarity to reference isolates from GenBank.”* Suggest revision to; “ *Homology comparison and phylogenetic tree analysis, utilizing the neighbor-joining tree method, were applied to assess the recent isolate’s similarity to reference isolates from GenBank*.” 

*“The recent virus exhibited 11 amino acid residue differences compared to the A/Layer/Indonesia/WestJava-04/2017 isolate.”*. please specify which gene is being referred to, or if this is the whole genome.  

Introduction

 There are 11 different subtypes of NA, please amend the second line of the introduction.

 “ *facilitates the easy spread and mutation of AIVs*”. Recommend removing ‘easy’ from this sentence.

Methods

 “ *After five years of vaccination with a homologous vaccine at the farm, a decrease in production and bird mortality occurred in February 2023.*” The authors may wish to consider publication of these potentially interesting data in another manuscript if they haven’t already. In this manuscript, information RE the vaccine used and the administration regimens should be included. This could be included in the introduction or results.

 “ *Samples consisting of brain, trachea, and oviduct organs pooled from deceased chickens*” More information would be helpful, perhaps in the results and/or as supplementary information.

- How many chickens?
- How many samples were tested and by which method?
- How did the chickens die? Were they symptomatic of influenza or other illnesses?
- Define the meaning of ‘pooled’ – were all organs from each chicken combined together in one sample or were different organs of the same type polled?
- How large is the farm in terms of numbers of chickens?

*“* *Tissue was combined with sterile phosphate-buffered saline from ThermoFisher (28348) pH 7.4 containing antibiotics (200 g/ml penicillin and 100 g/ml streptomycin) and then centrifuged at 1,000 g and 4 °C for fifteen minutes. The remaining supernatant was filtered through a 0.45 m membrane filter and injected intra-allantoically into 9-day-old SPF ECEs.* *”* Recommend modification to read; *“* *Tissue was combined with sterile phosphate-buffered saline* *(* *pH 7.4* *)* *from ThermoFisher (28348) containing antibiotics (200 g/ml penicillin and 100 g/ml streptomycin) and then centrifuged at 1,000 g and 4 °C for fifteen minutes. The remaining supernatant was filtered through a 0.45* *μ* *m membrane filter and injected intra-allantoically into 9-day-old SPF* *egg* *s.* *”* 

 How many samples were used to inoculate eggs?

*“* *The allantoic fluid that was collected was further extracted using a total RNA mini kit reagent from Geneaid®, Taiwan (RPD050).* *”*  Recommend modification to read; *“RNA* *was further extracted* *from t* *he allantoic fluid that was collected using a total RNA mini kit reagent from Geneaid®, Taiwan (RPD050).* *”*   

Results

*“A positive result was determined when the Ct value was less than 40 and displayed an amplification curve resembling that of the positive control. Conversely, a negative result was determined if the Ct value was 45 or higher, and no amplification curve resembling the positive control was observed. The results with a Ct value between 40 and 45 were classified as indeterminate or dubious.” *Perhaps this is too repetitive of the same information in the methods section?

*“The amino acid sequence at the cleavage site of the recent isolate is PSRSSR↓GLF, while the receptor binding site has the motif PWTNTLY (Table 1). Additionally, at position 217 on the left side of the receptor binding site (RBS), the recent isolate contained the amino acid M (methionine). It should be noted that this amino acid sequence is identical to A/Layer/Indonesia/WestJava-04/2017, which originated from the same farm.*” A bit more explanation of the significance of these would be useful.

Discussion

*“In conclusion, this study investigated the molecular characteristics of avian influenza virus subtype H9N2 in Indonesia, focusing on a farm in West Java province from 2017 to 2023.”* Is this correct? Was continued surveillance performed on this farm during this time period? If so, then these data should be included (such as numbers of samples collected, method of detection of influenza etc). Otherwise this sentence should be revised as it is a bit misleading.

Is the work clearly and accurately presented and does it cite the current literature?

Yes

If applicable, is the statistical analysis and its interpretation appropriate?

Not applicable

Are all the source data underlying the results available to ensure full reproducibility?

No source data required

Is the study design appropriate and is the work technically sound?

Yes

Are the conclusions drawn adequately supported by the results?

Partly

Are sufficient details of methods and analysis provided to allow replication by others?

Yes

Reviewer Expertise:

Influenza virus zoonosis and interspecies transmission

We confirm that we have read this submission and believe that we have an appropriate level of expertise to confirm that it is of an acceptable scientific standard, however we have significant reservations, as outlined above.

# Reviewer report: Reviewer response for version 1

**DOI:** 10.5256/f1000research.165593.r303169

**Published:** 2024-08-14

**Authors:** Amany Adel

**Affiliations:** 1 Animal Health Research Institute, Agriculture Research Center (ARC), Giza, Egypt

Thanks for giving me the chance to review this paper.

 I think it is an important study that supposed to present updated situation for H9N2 viruses in Indonesia, and I believe that this kind of study is very important and expressive to give an updated vision for the viruses around the world.

 Unfortunately, the study is not well developed and not well written, I think it needs to be more improved in writing and structuring.

 If you please take these notes in your consideration:

 1- The paper needs to be re-written well, the discussion is too long and needs to be more concise and targeted.

 2- The number of the collected samples are not clarified in the paper, also the epidemiological data is very leaked and does not represent the situation in Indonesia.

 3- I have a confusion in the classification of H9 in your study; your classification is not compatible with classification in the study of Silvia Carnaccini and Daniel R. Perez, 2020 (H9 Influenza Viruses: An Emerging Challenge), although you cited this paper in your study. please take a look in this study again and give explanation for this conflict.

 Best wishes.

Is the work clearly and accurately presented and does it cite the current literature?

Partly

If applicable, is the statistical analysis and its interpretation appropriate?

Not applicable

Are all the source data underlying the results available to ensure full reproducibility?

Yes

Is the study design appropriate and is the work technically sound?

Partly

Are the conclusions drawn adequately supported by the results?

Partly

Are sufficient details of methods and analysis provided to allow replication by others?

Partly

Reviewer Expertise:

virology, avian influenza, molecular diagnosis

I confirm that I have read this submission and believe that I have an appropriate level of expertise to confirm that it is of an acceptable scientific standard, however I have significant reservations, as outlined above.
